# LVC/MM: A Hybrid Linear Vibronic Coupling/Molecular Mechanics Model with Distributed Multipole-Based Electrostatic Embedding for Highly Efficient Surface Hopping Dynamics in Solution

**DOI:** 10.1021/acs.jctc.3c00805

**Published:** 2023-10-03

**Authors:** Severin Polonius, Oleksandra Zhuravel, Brigitta Bachmair, Sebastian Mai

**Affiliations:** † Institute of Theoretical Chemistry, Faculty of Chemistry, University of Vienna, Währinger Str. 17, 1090 Vienna, Austria; ‡ Vienna Doctoral School in Chemistry (DoSChem), University of Vienna, Währinger Str. 42, 1090 Vienna, Austria; § Research Platform on Accelerating Photoreaction Discovery (ViRAPID), University of Vienna, Währinger Str. 17, 1090 Vienna, Austria

## Abstract

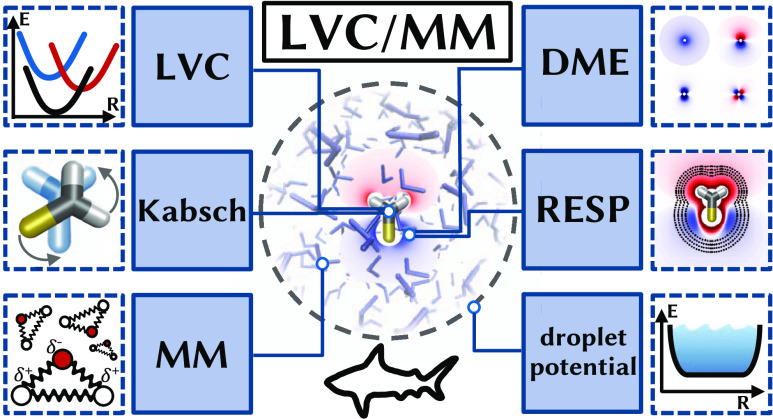

We present a theoretical framework for a hybrid linear vibronic coupling model electrostatically embedded into a molecular mechanics environment, termed the linear vibronic coupling/molecular mechanics (LVC/MM) method, for the surface hopping including arbitrary coupling (SHARC) molecular dynamics package. Electrostatic embedding is realized through the computation of interactions between environment point charges and distributed multipole expansions (DMEs, up to quadrupoles) that represent each electronic state and transition densities in the diabatic basis. The DME parameters are obtained through a restrained electrostatic potential (RESP) fit, which we extended to yield higher-order multipoles. We also implemented in SHARC a scheme for achieving roto-translational invariance of LVC models as well as a general quantum mechanics/molecular mechanics (QM/MM) interface, an OpenMM interface, and restraining potentials for simulating liquid droplets. Using thioformaldehyde in water as a test case, we demonstrate that LVC/MM can accurately reproduce the solvation structure and energetics of rigid solutes, with errors on the order of 1–2 kcal/mol compared to a BP86/MM reference. The implementation in SHARC is shown to be very efficient, enabling the simulation of trajectories on the nanosecond time scale in a matter of days.

## 1 Introduction

In computational studies on photoinduced processes in chemistry, biology, and materials sciences, nonadiabatic molecular dynamics (MD) simulations—MD simulations where more than one electronic state is considered—are nowadays an essential technique.^1−5^ The trajectory surface hopping (TSH) method^6−8^ is one of the widely adopted approaches for performing MD simulations of photoinduced processes. Using TSH, it is possible to simulate fundamental processes such as internal conversion (IC)^9^ or intersystem crossing (ISC).^10−12^ Therefore, TSH enables investigations into natural phenomena such as photoisomerization, photodissociation, and photocatalysis.^7^ These phenomena not only occur in isolated molecules in the gas phase but are also important in solution or even aggregated molecular systems—which could be subsumed as “complex systems”.^13^

Although TSH simulations (and other nonadiabatic dynamics techniques) can in principle elucidate essential details of the photoinduced process of molecular systems, they come at a sizable computational cost, especially for large systems. This cost arises because at every time step in TSH simulations, one needs to carry out excited-state electronic structure calculations, which yield the electronic potential energies, gradients, and nonadiabatic couplings. For typical small organic molecules, each such electronic structure calculation can take minutes to hours on modern computer hardware, scaling steeply with the system size. A simulation of a few picoseconds with adequate statistics requires thousands of electronic structure calculations multiplied by hundreds of trajectories, which leads to TSH projects that consume hundreds of thousands to millions of CPU hours.^3,14^ For this reason, simulations of complex molecular systems interacting with their environment quickly reach the limits of feasibility, especially when such systems exhibit interesting dynamical processes on longer time scales.

If all relevant electronic excitations of a large system are localized only in a small region, one typically employs hybrid quantum mechanics/molecular mechanics (QM/MM) techniques.^15−17^ In this way, the computational effort (dominated by the QM calculation) is focused on the relevant region, whereas the surroundings are treated at a lower cost. Despite the use of QM/MM, TSH projects can be computationally expensive—depending on the chosen method and QM region—and cheaper solutions are sought after. The latter include semiempirical methods in wave function or density functional theory (DFT) formulations,^18−20^ the construction of the potential energy surface through interpolation,^21,22^ the application of machine learning to predict energies, gradients, and other properties,^23,24^ and excited-state self-consistent field methods (Δ*S*CF).^25,26^

Another possibility of representing the coupled excited-state potential energy surfaces of molecules in an inexpensive fashion is given by vibronic coupling models, in particular, linear vibronic coupling (LVC) models. Historically, LVC was predominantly employed in multiconfigurational time-dependent Hartree and other quantum dynamics simulations.^27^ However, recently,^28^ LVC was combined with TSH in the framework of the SHARC (surface hopping including arbitrary couplings) package.^29,30^ The combination of TSH and LVC facilitates the description of the nonadiabatic gas-phase dynamics of rigid medium-sized molecules and transition-metal complexes with many states.^14^ While the use of LVC is restricted to rigid systems, it offers some advantages over the other low-scaling methods mentioned above. For example, the LVC model is highly efficient: it scales linearly with the number of degrees of freedom and quadratically with the number of states, and it does not employ any basis functions or an SCF cycle. It can be fitted to any reference electronic structure calculation, thus potentially providing good accuracy at a minimal cost. The model can properly describe conical intersections with the ground state, which is not possible with single reference methods. In the context of TSH, it can also provide analytical nonadiabatic coupling vectors, even if the reference method/software does not.

In order to capitalize on the advantages of LVC models in the context of complex systems, the main goal of the present contribution is to extend the LVC Hamiltonian to make it compatible with electrostatic embedding and hence suitable for the QM/MM method. This novel approach, termed LVC/MM, can be used for highly efficient nonadiabatic dynamics simulations of stiff molecules embedded in an environment. In order to do so, we combine several previously published techniques.

We use a QM–MM interaction Hamiltonian that is based on an electrostatic embedding^15^ scheme, where the MM charge distribution is represented by fixed point charges and directly affects the QM electronic states. As there are no explicit electronic wave functions in the LVC models, we represent all relevant electron and transition densities with static parameters. In order to represent these densities accurately—especially anisotropic densities like lone pairs, π-systems, or out-of-plane transition densities—we use an atomic multipole expansion^31,32^ analogous to the one used in the AMOEBA force field.^33^ These atomic multipoles are defined in a diabatic basis (the same basis is used to set up the LVC Hamiltonian), which has previously been suggested to enable a good charge representation even when the nuclear coordinates change.^34^

In order to obtain multipole parameters for all electronic states and transitions during the parameter generation of the LVC model, we employ the restrained electrostatic potential (RESP) method.^35^ In RESP, atomic point charges are fitted to reproduce the ESP around the molecule, which ensures an accurate representation of electrostatic interactions with the surrounding solvent.^36^ Alternative methods to obtain (multipolar) atomic charges are the distributed multipole analysis of Stone et al.^32,37^ (as used in AMOEBA^33^) or various population analysis methods,^38−41^ although the latter seem less suitable for reproducing the ESP.^35,42^ In the present work, we extend the RESP method to fit not only point charges but also the higher multipole terms for both electron and transition densities.

In the following, we first describe the theory behind the essential ingredients of LVC/MM: the basic equations of the LVC model, achieving translational and rotational invariance for the LVC model, electrostatic embedding QM/MM, the QM–MM Coulomb interaction term, and finally the extended RESP procedure. Subsequently, we present the application of LVC/MM to the case of thioformaldehyde (CH_2_S) in water. This molecule was used before as a testbed to compare electronic structure methods and decoherence schemes in TSH.^43,44^ Note that thioformaldehyde is unstable under normal conditions (owing to fast oligomerization), but it is a useful model as the smallest molecule with a thiocarbonyl bond. Its nonadiabatic dynamics is governed by its  (S_1_–T_2_) gap, which in the gas phase is too large to allow ISC.^43^ Here, we investigate how the energies of the electronic states are influenced by the aqueous environment and especially how the  (S_1_–T_2_) gap is affected. Focusing on these aspects, we scrutinize the performance of LVC/MM versus the reference QM/MM simulation at the BP86/def2-SVP level of theory in several aspects.

## 2 Theory

In this section, we provide all of the working equations for the LVC/MM approach.

### 2.1 Linear Vibronic Coupling

In a general vibronic coupling model,^14,27,45^ the diabatic electronic Hamiltonian matrix **V** (called LVC Hamiltonian here) is constructed from matrix elements *V*_*ij*_, for all pairs of diabatic states *ij*, as
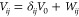
1where δ_*ij*_ is the Kronecker delta, *V*_0_ is the reference potential, and *W*_*ij*_ is an element of the vibronic coupling matrix. *V*_0_ is usually approximated as a harmonic potential^45^ and is written in terms of the dimensionless mass–frequency-scaled normal coordinate vector **Q** as
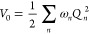
2where ω_*n*_ are the normal-mode frequencies. The components *Q*_*n*_ of **Q** for every normal mode *n* can be written as
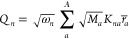
3Here, matrix **K** contains the orthogonal and mass-weighted normal-mode vectors for *A* atoms and **r̅** = **r** – **r**_0_ is the Cartesian displacement vector from the reference geometry. In total, the reference potential *V*_0_ can be calculated using eqs 2 and 3 from the current Cartesian coordinates **r**, the reference geometry **r**_0_, the transformation matrix **K**, the atomic masses **M**, and the normal-mode frequencies **ω**.

In an LVC model, the elements of **W** are expanded in a Taylor series around **Q** = 0 up to the linear terms
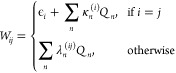
4with the vertical excitation energies ϵ_*i*_, and the intrastate and interstate vibronic coupling constants κ_*n*_^(*i*)^ and λ_*n*_^(*ij*)^.

The coupling constants κ_*n*_^(*i*)^ and λ_*n*_^(*ij*)^ are the first derivatives of the elements of the coupling matrix **W**, which is generally constructed in a diabatic basis. Thus, the values of the coupling constants cannot be extracted directly from the electronic structure calculations performed in the adiabatic basis. Therefore, in the construction of LVC models,^45^ it is typically assumed that at the reference geometry (**Q** = **0**), the adiabatic and diabatic states coincide. This allows evaluating the κ_*n*_^(*i*)^ and λ_*n*_^(*ij*)^ as
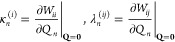
5Hence, the κ_*n*_^(*i*)^ are accessible from the Cartesian gradient of the adiabatic state *i* at **Q** = 0 after transformation into normal-mode coordinates. Likewise, λ_*n*_^(*ij*)^ can be obtained from nonadiabatic coupling (NAC) vectors.^28^ As NAC vectors are only implemented for a few electronic structure methods, according to Fumanal et al.,^45^ λ_*n*_^(*ij*)^ can alternatively be computed via finite differences of the excited-state energies after diabatization using wave function overlaps.

TSH simulations in SHARC typically employ electronic structure data (energies, gradients, couplings) in the eigenbasis of the molecular Coulomb Hamiltonian (MCH)^28,29^ (often called the adiabatic basis). Therefore, we transform the diabatic LVC Hamiltonian **V** into the MCH eigenbasis and then calculate the energies and gradients. In detail, we obtain **Q** from the coordinates via eq 3 and calculate *V*_0_ via eq 2. Subsequently, we calculate **W** (eq 4) and with it construct **V** via eq 1. The transformation to the MCH basis is done via the eigendecomposition
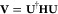
6where **H** is the Hamiltonian in the adiabatic basis, and **U** is a unitary transformation matrix. Analogous to many electronic structure methods, SOCs are only evaluated after diagonalization (here, by transforming diabatic SOCs η into the MCH basis) and added to the Hamiltonian as
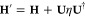
7Likewise, gradients  are transformed as
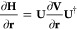
8where  is obtained from a coordinate transformation (following eq 3) of . Note that eq 8 also produces nonadiabatic coupling vectors.^28^

### 2.2 Rotationally and Translationally Invariant LVC

The parametrization of the LVC Hamiltonian is done in terms of the normal-mode vector **Q**, which is obtained from Cartesian displacement vector **r̅** and reference geometry **r**_0_ (eq 3). Here, the computation of the correct displacement vector requires that the current geometry **r** is aligned properly with the reference geometry **r**_0_. For TSH simulations in the gas phase, alignment is typically not a problem in the absence of roto-translational motion. However, in a system consisting of the LVC subsystem and environment, the position and orientation of the LVC subsystem can change due to diffusion processes.

For this reason, LVC/MM requires that the normal-mode vector **Q** be computed in a rotationally and translationally invariant fashion. This is achieved by superimposing (i.e., aligning) the current geometry **r**_S_ onto **r**_0_ before computing **Q** with eq 3. In other words, we will switch the frame of reference and the coordinate system from the one in solution “S” to the one of the reference geometry “0”. First, we calculate the centers of coordinates **c**_S_ and **c**_0_ of **r**_S_ and **r**_0_ as
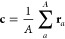
9Next, we shift the coordinates of all atoms *a* to the origin by subtracting to obtain **r**_0,*a*_ – **c**_0_ = **r̃**_0,*a*_ and **r**_S,*a*_ – **c**_S_ = **r̃**_S,*a*_. Using the Kabsch algorithm,^46^ we determine the optimal rotation matrix **T**^rot^, which transforms the current coordinates such that the root-mean-square distances with respect to the reference coordinates are minimized. The first step in this algorithm is the construction of a 3 × 3 cross-covariance matrix **K**_S0_ and the computation of its singular value decomposition
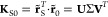
10where **r̃**_S_ and **r̃**_0_ are *n*_atom_ × 3 matrices. We compute the optimal rotation matrix **T**_S0_^rot^, which transforms from the solution frame “S” to the reference frame “0″, as
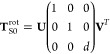
11where *d* is the determinant of **UV**^*T*^. Finally, we transform **r**_S_ into the reference frame **r**_S(0)_ as
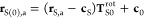
12and subsequently calculate **Q** from eq 3 using **r**_S(0)_ (i.e., in the reference frame).

This procedure enables the computation of the LVC Hamiltonian and thus energies of the LVC model in a rotationally and translationally invariant fashion. However, equivariant properties—like gradients or dipole moments, also need to be transformed back into the simulation frame. Dipole moments are simply rotated using the transpose of **T**_0S_^rot^. The gradients of the diabatic LVC Hamiltonian matrix elements can be expanded as
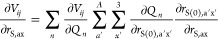
13Here, the partial derivatives  on the right-hand side are given by differentiation of eqs 2 and 4, and the partial derivatives  with respect to each atom *a* and direction *x* are given by differentiation of eq 3. The third term  could in principle be obtained by differentiation of the Kabsch algorithm, although this is rather complicated (involving the differentiation of a singular value decomposition). Instead, we evaluate this term numerically, which is still efficient as it depends on only the number of atoms in the QM region (i.e., atoms considered in the LVC model).

### 2.3 Electrostatic Embedding

In a QM/MM calculation, the total molecular system is split into two parts; one that is described quantum-mechanically and the other by molecular mechanics.^15,47^ In the widely used electrostatic embedding formalism,^15^ the total energy of the system *E*_QM/MM_ can be constructed from the energies of three different calculations as
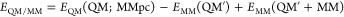
14Here, *E*_QM_(QM; MMpc) is the energy of the QM region at the QM level including the interaction of the point charges of the MM region with the QM region. *E*_MM_(QM′ + MM) is the energy of the entire system at the MM level but with the point charges of the QM region set to zero. *E*_MM_(QM′) is the energy of the QM region at the MM level also with the point charges of the QM region set to zero. Setting the point charges of the QM region to zero at the MM level avoids double-counting of the Coulomb interaction between QM and MM atoms, which is already considered in the QM calculation.

In LVC/MM, the Coulomb interaction between QM and MM atoms (a component of *E*_QM_(QM; MMpc)) is considered by extending the LVC Hamiltonian in eq 1 by interaction matrix **X**
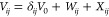
15The interaction matrix elements *X*_*ij*_ formally consist of the electrostatic interaction between the point charges of the MM atoms *b* and the QM region’s electronic densities ρ^(*ij*)^ and nuclei *a*
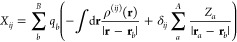
16Here, *q*_*b*_ are the point charges of the MM atoms, ρ^*ij*^ is the electronic density of state *i* of the QM region (transition density between *i* and *j* for *i* ≠ *j*), and *Z*_*a*_ is the nuclear charge of atom *a*. Here, the diagonal elements *X*_*ii*_ produce solvent-induced energy shifts of the diabatic states, whereas the off-diagonal elements *X*_*ij*_ generate solvent-induced state mixing.

In an LVC calculation, we consider it unfavorable to work with the actual electron densities ρ^*ij*^, as their representation (in terms of basis functions and density matrices) and evaluation (of the electrostatic interaction integrals) would present serious bottlenecks in the computation. Hence, the electrostatic potential (ESP) at a point **r**_*b*_ exerted by ρ^(*ij*)^
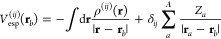
17needs to be approximated, as detailed in the next section.

### 2.4 Distributed Multipole Expansion

The distributed multipole expansion (DME) is a Tailor expansion of the Coulomb potential around the nuclear coordinates as centers.^37^ Higher-order terms of the Tailor expansion capture anisotropies in the short-range electrostatics of the electronic density; we include terms up to the second order, i.e., up to quadrupoles on each atom. This allows representing, e.g., out-of-plane transition densities in planar molecules or lone pair electron clouds.

The ESP at a point in space **r***_g_* arising from all QM atoms *a* for a pair of states *ij* (see eq 17) can be approximated as a Taylor expansion
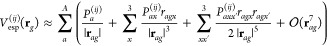
18Here, *P*_*a*_^(*ij*)^ is an atomic monopole parameter representing density ρ^*ij*^ and nuclear charge *Z*_*a*_ on atom *a*, equivalent to the partial charges typically used in most MM force fields.^33,48,49^ Analogously, *P*_*ax*_^(*ij*)^ is a corresponding dipole term with the three Cartesian axes indexed by *x*, and *P*_*axx*′_^(*ij*)^ is a corresponding quadrupole term in the direction *xx*′. *r*_*agx*_ is a component of vector **r**_*ag*_, the difference vector between the coordinates of atom *a* and the point in space **r**_*g*_. This formula can be rewritten in terms of a sum over scalar products between a tensor **P**^(*ij*)^ containing multipole terms and a geometric tensor **T** containing all of the corresponding prefactors. For clarity, we use the index *p* to enumerate the different multipole elements on an atom *a* (in the order monopole, *x*, *y*, *z*, *xx*, *yy*, *zz*, *xy*, *xz*, *yz*). Then, the ESP is
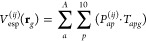
19and the interaction elements of the LVC Hamiltonian become
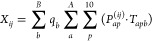
20

In order to compute the total LVC/MM gradients and nonadiabatic coupling vectors, we require the gradients of the interaction elements. The derivative with respect to the position of an MM atom *b* is
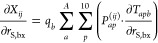
21The gradients of the LVC atoms *a* are slightly more involved
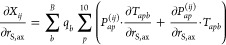
22For both equations, the multipole parameters *P*_*ap*_^(*ij*)^ are first rotated from the reference frame (in which they are defined) to the system frame. The derivative  arises because the rotation of the parameters depends on the position of the LVC atoms that enter the Kabsch algorithm (see the discussion of eq 13). This derivative can be computed numerically.

Because the LVC model based on eq 15 is defined on the diabatic electronic basis, the DME parameters *P*_*ap*_^(*ij*)^ also represent the electronic (transition) densities of the diabatic states. In this regard, the electrostatic LVC/MM model is related to the diabatic charge matrix approach of Park and Rhee,^34^ who showed that diabatic charges retain validity for the same diabatic pair of states at different geometries. Although the use of constant diabatic charges in the LVC/MM model constitutes an approximation, we assume that this approximation is valid for the range of geometries for which the LVC model itself is valid. Thus, LVC/MM using constant diabatic charges *P*_*ap*_^(*ij*)^ should only be applied for relatively rigid systems that stay close to the reference geometry.

### 2.5 Restrained Electrostatic Potential Fit

The last key ingredient for the LVC/MM model is an algorithm to obtain the set of all DME parameters *P*_*ap*_^(*ij*)^ from an electron density ρ^*ij*^. To this end, we extend the restrained electrostatic potential (RESP) method^35^ to atomic multipoles. We chose RESP because it is derived to reproduce the molecular ESP, which is precisely the term we are interested in for electrostatic embedding (eq 16). It has been shown that RESP produces more accurate electrostatics compared to other population analysis methods, especially if buried atoms are involved.^36^

The underlying idea of the RESP method^35^ is to define a grid of points *g* around the molecule,^50^ compute the ESP arising from the electron density ρ^*ij*^ (and nuclei) at each grid point, and then optimize the atomic charges to best reproduce the ESP at all grid points.

In order to generate the fitting grid, we employ the Merz–Singh–Kollman scheme.^50−52^ First, for each atom, a set of spherical shells is generated, with radii *R*_*i*_ that are multiples of the atom’s van-der-Waals (vdW) radius *R*_vdW_. The shells are equidistant with radii
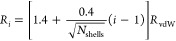
23where the closest shell is at 1.4*R*_vdW_ and *i* = 1, ···, *N*_shells_ enumerates the shells. This scheme is taken from Gaussian^53^ and reproduces the original Merz–Singh–Kollman scheme with shells of 1.4, 1.6, 1.8, and 2.0 times *R*_vdW_ for *N*_shells_ = 4. Second, each shell is discretized into a set of points by using a spherical surface quadrature. We use the Lebedev quadrature^54^ because it is highly symmetric (point group *O*_*h*_) and thus ensures that the fitted multipoles adhere to the symmetry of the molecule for most point groups. A user-defined point density is used to determine the order of the Lebedev quadrature for each shell. Third, as in the Merz–Singh–Kollman scheme,^50−52^ all grid points are removed whose *R*_vdW_-scaled distance to another atom is smaller than to the atom they are centered on. This produces the final set of grid points used for fitting.

Once the grid is set up, the ESP *V*_esp_^(*ij*)^ at all grid points **r**_*g*_ is calculated using eq 16. In our present implementation, we use the PySCF package^55,56^ to evaluate the integrals over ρ^(*ij*)^ as
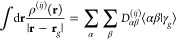
24Here, ⟨αβ|γ_*g*_⟩ is a three-center-two-electron integral over the atomic orbitals α and β and a very tight (exponent 10^16^) *s* function γ_*g*_ centered at **r**_*g*_. *D*_αβ_^(*ij*)^ is an element of the density matrix for state pair *ij*.

The required density matrices **D**^(*ij*)^ are obtained from the same electronic structure calculation that was used to derive the LVC parameters (ε’s, κ’s, λ’s). For wave function-based methods (e.g., CASSCF), the matrices for all state pairs *ij* can typically be obtained directly from an electronic structure software. For linear-response methods, only the ground-state density matrix **D**^00^ and the ground-to-excited-state transition density matrices **D**^0*i*^ (*i* > 0) are automatically available. The relaxed excited-state densities **D**^(*ii*)^ (*i* > 0) require additional computational effort,^57^ and the excited-to-excited-state transition density matrices **D**^(*ij*)^ (*i*, *j* > 0) can be approximated^58^ as
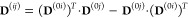
25

In the last step, we perform the actual RESP fit. We first compute the geometric tensor with elements *T*_*apg*_ based on the atom and grid positions and then
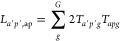
26which are the elements of the matrix **L** with composite index *ap*. Second, for each state pair *ij*, we compute the vector **Y** with elements
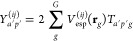
27Then, we can obtain the desired DME parameter set **P**^(*ij*)^ (i.e., all elements *P*_*ap*_^(*ij*)^) from the equation
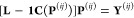
28where **1** is a unit matrix and **C**(**P***^ij^*) is the vector collecting the restraint elements
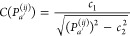
29Here, *c*_1_ and *c*_2_ are constants that define the hyperbolic restrictions of the RESP method. The full derivation is given in the Appendix.

Since **C**(**P**^(*ij*)^) depends on the fitted charges, eq 28 has to be solved iteratively. The initial guess for **P**^(*ij*)^ is obtained using eq 28 without restraints. Subsequently, the constraint is evaluated and added to the diagonal of **L**, the linear equation system is solved, and new **P**^(*ij*)^ are computed repeatedly until self-consistency.

The equations given above are general in the sense that one can fit all of the multipole orders at once. However, in practice, we fit the parameters order by order; i.e., we first fit the monopoles (recovering standard RESP charges). We subtract the ESP of the monopoles from the reference ESP and fit the dipoles to this residual. Finally, we repeat the procedure again for the quadrupoles. Here, we can employ different restraint parameters *c*_1_ and *c*_2_ for different multipole orders, typically with stronger restraints for higher orders. In addition to the “soft” restraints described above, we utilize “hard” constraints in our fit procedure. In this way, we ensure conservation of the total molecular charge (via a constraint acting on the monopoles) and ensure that the quadrupoles on each atom are traceless. The constraints are implemented by adding additional equations to the linear equation system represented by eq 28.^59^

## 3 Computational Details

In order to demonstrate that LVC/MM can reproduce QM/MM results and to verify that our implementation is correct, we validated several aspects of the model and simulation results against a reference. Although the method is intended to be used in TSH, in the present form, we carry out validation of the simulation results mostly for a single state, to focus on the solute–solvent interactions and the accuracy of the method. The investigation of the coupled solvent–solute excited-state dynamics of CH_2_S via a TSH simulation will be presented in a separate work.

First, we constructed a set of LVC model parameters, including multipole charges for thioformaldehyde. Second, we constructed the full molecular system, thioformaldehyde in water, and performed preliminary equilibration using AMBER.^49^ Subsequently, we ran both DFT/MM and LVC/MM simulations with SHARC,^29,60^ from which we extracted snapshots to compute solvent distributions. Additionally, we performed single-point calculations on the snapshots with DFT and LVC in the gas phase and solution to scrutinize the agreement of LVC with its reference.

### 3.1 LVC Model

The LVC model for thioformaldehyde was constructed at the BP86/def2-SVP^61−63^ level of theory. We employed a combination of calculations using Gaussian 16^53^—which provides convenient access to all (relaxed) density matrices needed to fit the DME—and ORCA 5,^64^ which provides spin–orbit couplings and is better supported in SHARC for efficient QM/MM trajectories. To ensure consistent results, we set the inputs for both programs to use the same definition of the BP86 functional with the VWN5^65^ local correlation. Excited states were computed within the Tamm–Damcoff approximation.

The reference harmonic oscillator *V*_0_ (eq 2) was parametrized from a ground-state optimization and frequency calculation in ORCA. The vibronic coupling parameters (eq 4) were obtained via finite differences^45^ using excited-state calculations in Gaussian. We computed four states (S_0_, S_1_, T_1_, and T_2_) where we set a very tight convergence threshold (“conver = 9”) and used the “superfine” grid. The various LVC parameters were computed as previously described.^28,45^

At the reference geometry (S_0_ minimum), all relaxed-state densities and transition densities were extracted from the Gaussian output to fit the DME for each state pair. We used the original Merz–Singh–Kollman scheme^50−52^ with four shells, the corresponding vdW radii,^50^ and Lebedev quadratures^54^ with a density of 10 points/Å^2^, for a total of 6340 grid points. To prevent overfitting, the *c*_2_ restraint parameter in eq 33 is increased for higher-order multipoles, using values of 0.0005, 0.0015, and 0.003 (in units of elementary charge) for monopoles, dipoles, and quadrupoles, respectively.

All LVC model parameters are reproduced in Sections S1 and S2 and Tables S1–S4.

### 3.2 System Preparation

The system was prepared using tools from the Amber program package.^49^ Thioformaldehyde was solvated in a 15 Å truncated octahedron box of 1091 TIP3P^66^ water molecules. Using periodic boundary conditions, a 2 fs time step, and GAFF2 parameters^67^ for thioformaldehyde, the system was then optimized for 1000 steps, heated for 50 ps to 300 K (NVT ensemble), and equilibrated for 50 ns at 300 K and 1 bar (NpT ensemble). The final coordinates and velocities were reimaged into the original box and converted to the SHARC initial condition format.^68^

### 3.3 SHARC Simulations

Starting from this set of initial conditions, we performed four 1 ns trajectories in S_0_ using a local development version of SHARC 3.0.^29,60^ One trajectory was run with BP86/def2-SVP/MM using ORCA 5,^64^ while the other three used LVC/MM, using the level of theory/parameters described above, respectively. Out of the three LVC/MM trajectories, one was restricted to only monopole charges (labeled as LVC-DME0/MM henceforth), the second was using monopoles and dipoles (LVC-DME1/MM), and the last one was using terms up to quadrupoles (LVC-DME2/MM). All trajectories used a 2 fs time step (500,000 steps). The TIP3P water molecules and the two C–H bonds of thioformaldehyde were constrained to their equilibrium values using the RATTLE algorithm implemented in SHARC 3.0.^69,70^ The temperature was set to 293 K via the Langevin thermostat^71^ implemented in SHARC.^72^

SHARC^29,60^ is not able to perform simulations with periodic boundary conditions. Therefore, we utilized the droplet potential of Sankararamakrishnan et al.^73^ to keep the water droplet spherical, with a radius of about 19.8 Å. An additional tethering potential was applied to thioformaldehyde to keep it centered within the droplet. Both potentials use the same flat-bottom harmonic potential
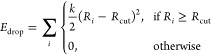
30where *R*_*i*_ is the distance of a given atom from the origin, *k* is the force constant, and *R*_cut_ is a threshold radius. For the droplet potential, the sum included all atoms, and *R*_cut_ was 15.86 Å. For the tethering potential, the sum included only the thioformaldehyde atoms, and *R*_cut_ was 2 Å. The force constant was 0.81 μ*E*_h_/Bohr in both cases.

As shown in eq 14, at each time step, we performed three calculations: one with ORCA or LVC, and two at the MM level. MM calculations were performed with a newly developed SHARC interface to the OpenMM package.^74^ As input, this interface used the parameter/topology files from Amber, where the thioformaldehyde charges were set to zero. The three calculations were then combined in a newly developed general QM/MM interface in SHARC.

From each of the four 1 ns trajectories, the first 50 ps was discarded to allow for reequilibration. From the remaining 950 ps, snapshots were taken every 100 fs, giving four sets of 9502 snapshots for further analysis. The solvation structure obtained was investigated by using radial distribution functions (RDF) with a bin width of 0.05 Å. Furthermore, we computed the solvent distribution around thioformaldehyde using three-dimensional (3D) histograms of the O and H atoms of water, with a grid spacing of 0.5 Å. RDFs and 3D distributions were calculated from the entire data sets (9502 snapshots each) using cpptraj from AmberTools.^49^

In order to further compare LVC/MM to its reference method, we performed single-point energy calculations on all snapshots of the DFT/MM trajectory (either with water or after removing all waters). These single-point calculations were performed only at the LVC-DME2 level, not at the more approximate LVC-DME0 or LVC-DME1 levels.

## 4 Results and Discussion

In this section, we first summarize the electronic structure of the relevant states of thioformaldehyde. Subsequently, we discuss the quality of the RESP fits in reproducing the ESP of the molecule’s states in its vicinity. Finally, we discuss the results of the DFT/MM and LVC/MM trajectories and the computed solvation structure.

### 4.1 Electronic Structure of Thioformaldehyde

As discussed in a previous work,^43^ thioformaldehyde has four electronic states that are relevant at low energies (<3.5 eV). These are the closed-shell ground state (S_0_), the singlet ^1^nπ* state (S_1_), the triplet ^3^nπ* state (T_1_), and the triplet ^3^ππ* state (T_2_). High-level multireference calculations^43^ place their vertical excitation energies at 2.22, 1.96, and 3.44 eV, respectively, while our present BP86/def2-SVP calculations give values of 2.16, 1.54, and 3.34 eV. Higher states only appear above 5.5 eV with BP86. Note that these energies are given only for completeness; here, we are only interested in reproducing the BP86 results with LVC, without extensive concern for the accuracy of BP86.

**Figure 1:**
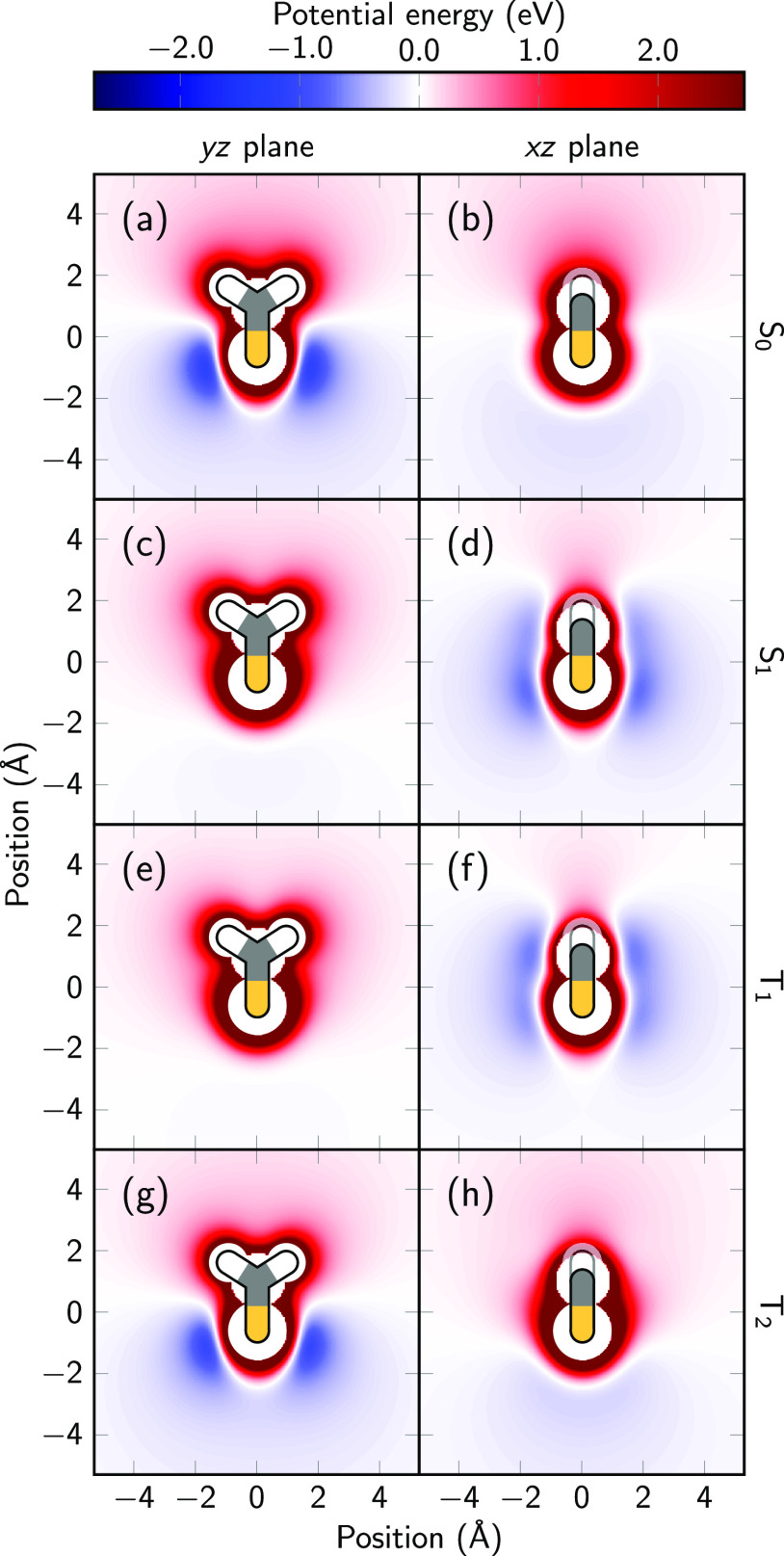
Electrostatic potential energy in the *yz* and *xz* planes of the first four excited states of thioformaldehyde for a unit positive charge computed with BP86/def2-SVP.

The four electronic states have different electron distributions, owing to their different occupations of n, π, and π* orbitals. These different electron densities give rise to different ESP energies (proportional to the ESP) around the molecule, as shown in Figure 1. The S_0_ state (panels a and b) shows a clear dipole moment in the *z* direction (negative near the S atom, positive near the H atoms). The most interesting feature of the S_0_ ESP are the two deep minima close to the S atom, which are located in the molecular plane; there are no such minima above/below the molecule. These two minima arise from the high electron density of the S atom’s lone pair orbital *n*.

The S_1_ (panels c and d) and T_1_ (e and f) states show very similar ESPs, which are distinctively different from the S_0_ ESP. Both S_1_ and T_1_ are nπ* states, where one electron is removed from the in-plane lone pair and added to the out-of-plane π* orbital. Consequently, the in-plane minima in the ESP vanish, and two shallow, somewhat delocalized minima above and below the C–S bond appear (i.e., in the *xz* plane). The shift of electron density from the S to the C atom also reduces the dipole moment considerably. Finally, the T_2_ state (panels g and h) has a ππ* configuration, and its electron density does not differ significantly from that of the S_0_ state. Therefore, its ESP is very similar to that of S_0_, including the two in-plane minima arising from the doubly occupied lone pair orbital.

We note that all states are strongly repulsive near the nuclei, where the positive probe charge penetrates the electron density. It would be desirable that the fitted DME for each state can reproduce all of the relevant features of the ESP from the BP86 calculations: the dipole far field, the attractive minima, and also the repulsive features.

### 4.2 Quality of the Fitted DME

**Figure 2:**
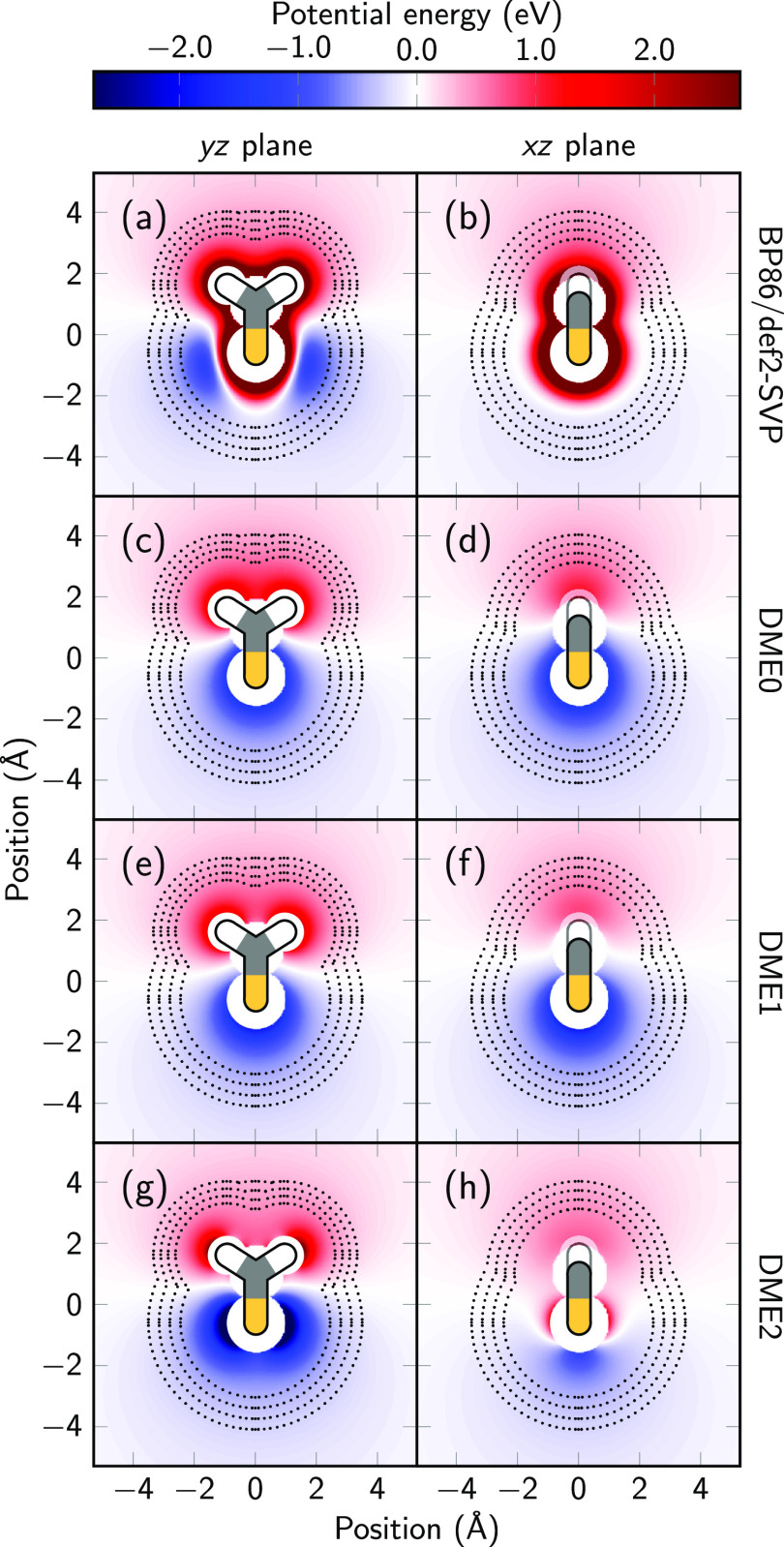
Electrostatic potential energy in the *yz* and *xz* planes of the S_0_ for BP86/def2-SVP compared to DMEs of order 0, 1, and 2, labeled DME0, DME1, and DME2, respectively. The black dots indicate the position of the fitting grid points used in the RESP procedure.

In Figure 2, we present the ESP energies for the S_0_ obtained from the reference BP86 (top row) and from three DMEs of orders 0, 1, and 2 (second–fourth rows, labeled DME0, DME1, and DME2 from here). The color scale and orientation of the molecule are identical to those in Figure 1. Additionally, we show the fitting grid points (black dots) that are located in the plot planes.

The ESP shown in panels (a and b) is the same as that in Figure 1. One can nicely see the distribution of the fit grid points, which avoid the highly repulsive area where the electron density is penetrated and densely surrounds the molecule. In panels (c and d), we show the ESP plots obtained by using only the monopole charges fitted with RESP to the electron density of S_0_ at the BP86/def2-SVP level of theory. As shown in Table 1, these are nearly identical to the RESP charges obtained with Gaussian and antechamber from AmberTools, using identical parameters for shell generation and point density. The minimal remaining differences arise because Gaussian generates the grid points by using a different quadrature.

**Table 1:** Fitted Monopole Charges of S_0_ at BP86/def2-SVP Level of Theory Using Gaussian and Amber versus Our Implementation

atom	Gaussian + antechamber	present work
C	–0.06743	–0.06637
S	–0.13338	–0.13392
H	*+*0.10040	*+*0.10015
H	*+*0.10040	*+*0.10015
RMSD		*+*0.00062

While our fitted monopoles reproduce RESP charges, they are only partially able to reproduce the details of the electrostatics of the molecule. The dipole moment is relatively well-reproduced (BP86/def2-SVP: 1.573 D; DME0:1.619 D). However, it is not possible to represent the anisotropy of the ESP around the molecule—the attractive interaction is too weak at the fit points in the molecular (*yz*) plane and too strong in the *xz* plane. The introduction of dipole terms in panels (e and f) does not fundamentally change this behavior. The dipole moment increases slightly to 1.647 D, but we consider this to be still in good agreement with the reference because our fitting grid is not intended to probe the far-field ESP of the molecule. Only when adding the quadrupole terms (panels g and h) do we obtain increased attraction and two minima in the molecular plane as well as a much reduced attraction above/below the molecule.

Figure 2 also clearly shows that our DME approach cannot describe in any way the repulsive region very close to the nuclei where the probe charge penetrates the electron density. This is because the DME approach is based on numerically efficient pointlike multipoles, whereas the electron density is a distribution. Fortunately, in typical MD simulations, atoms cannot come close enough to penetrate deeply into another molecule’s electron density due to Pauli repulsion. In MD simulations, Pauli repulsion is commonly represented by a part of the Lennard–Jones interaction.

**Figure 3:**
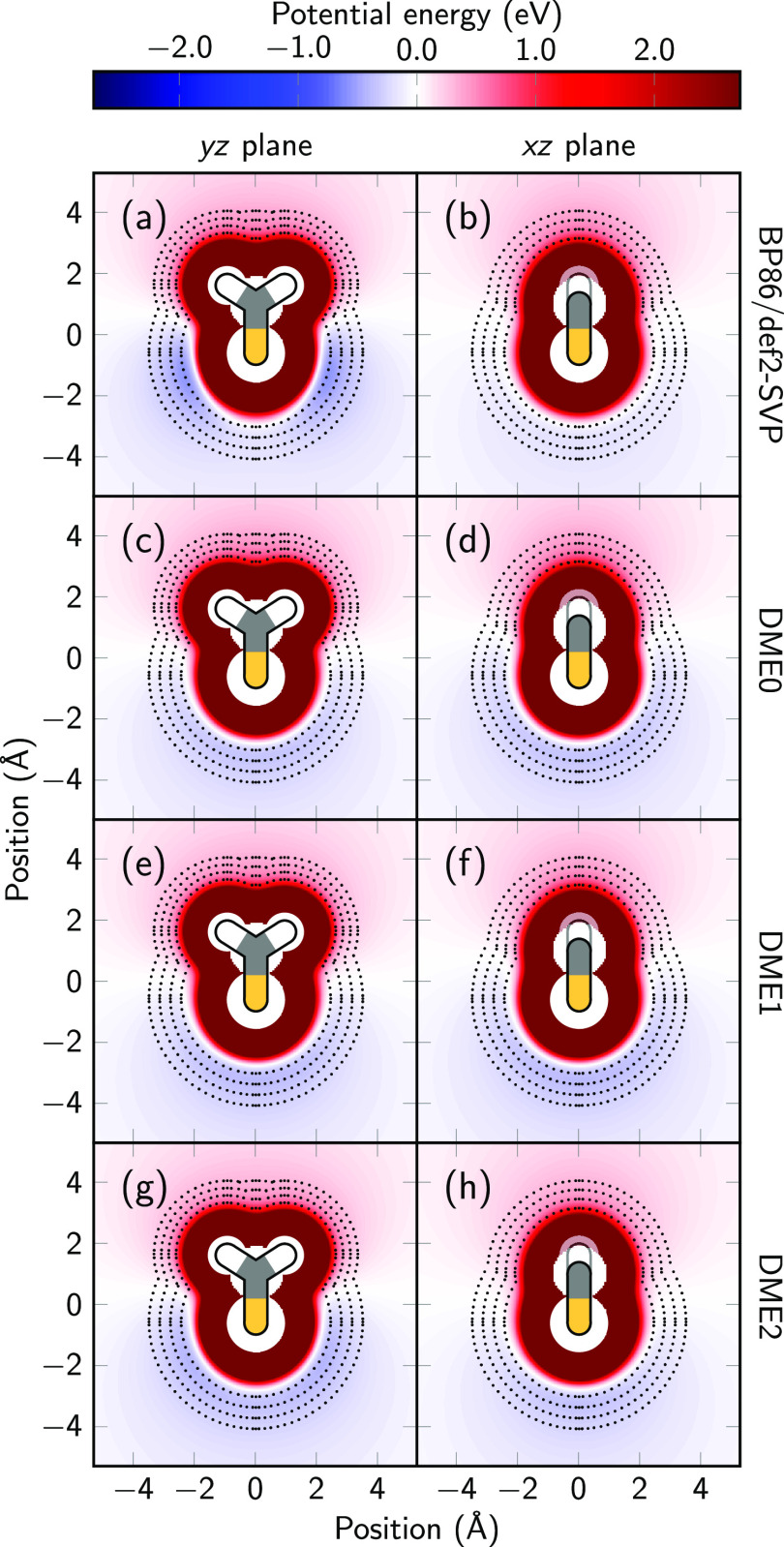
Electrostatic plus Lennard–Jones potential energy in the *yz* and *xz* planes of the S_0_ for BP86/def2-SVP compared to DMEs of orders 0, 1, and 2 labeled DME0, DME1, and DME2, respectively. Lennard–Jones energies are computed using GAFF2 parameters. The black dots indicate the position of the fitting grid points used in the RESP procedure.

In Figure 3, we show the sum of electrostatic and Lennard-Jones energies, where the latter will be very repulsive close to the nuclei, covering the region where the DME gives inaccurate results. Note that the Lennard–Jones interaction is shown for a probe H atom with GAFF2 parameters,^75^ which has a very small vdW radius. For larger atoms, an even larger region around the nuclei would be blocked. It can be nicely seen in Figure 3 that the Merz–Singh–Kollman scheme^50^ produces a sensible grid, where the innermost shell straddles the repulsive Lennard–Jones potential and the outer shells sample positions where it is very likely to encounter other atoms.

**Figure 4:**
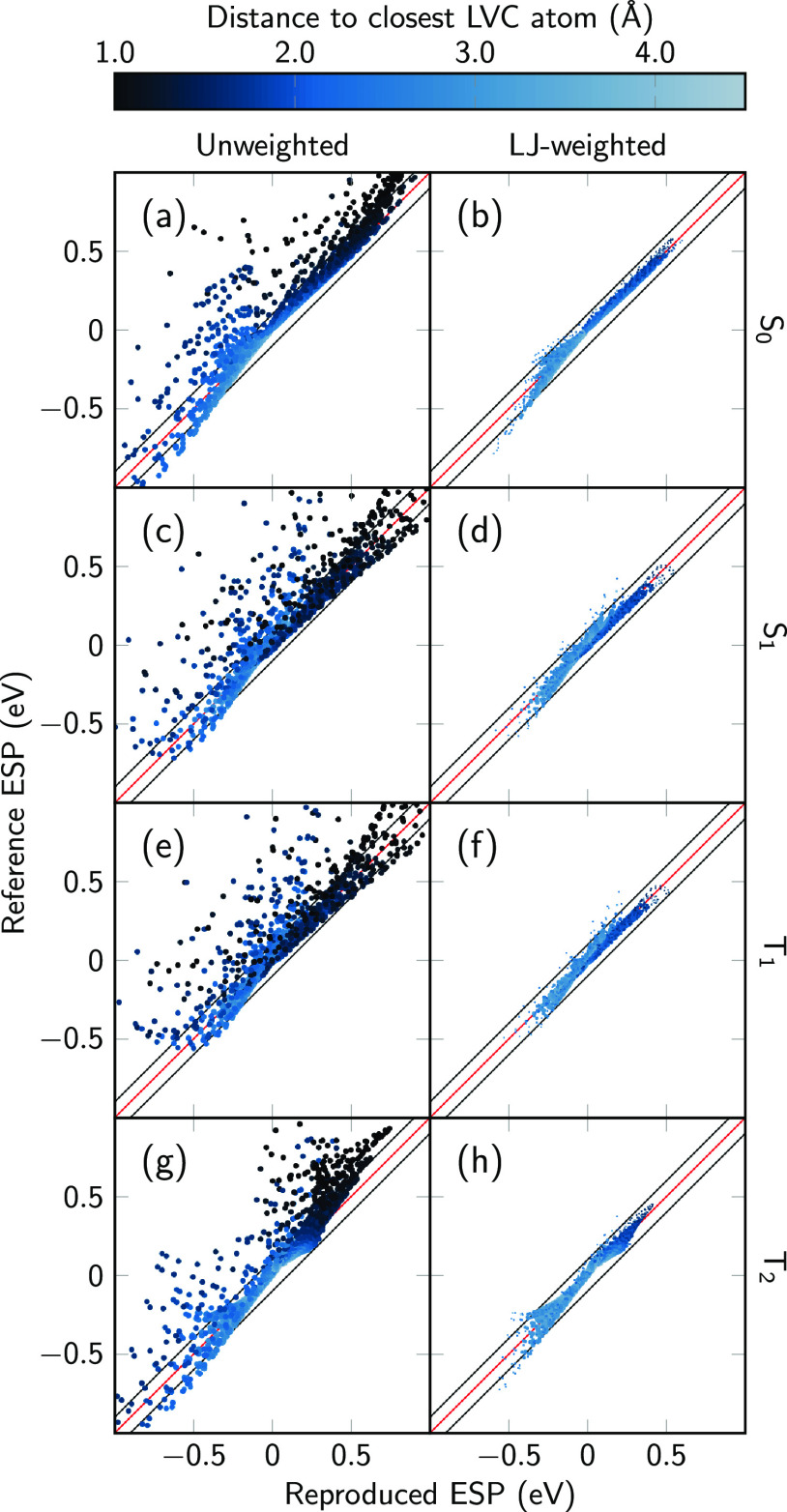
Correlation plots comparing the reference ESP energy (BP86/def2-SVP, vertical axes) with the ESP energy produced by the DME up to 2 order (horizontal axes). The plots use a test grid that is different from the fitting grid, with 6482 points in shells with radii 1.0–2.5 times the vdW radii. The color of the points indicates the distance to the closest atom. The red and two black lines indicate deviations of 0 and ±0.1 eV, respectively. On the left, all points are shown with equal point size, whereas on the right, the point size is scaled by the Boltzmann weight of the Lennard-Jones interaction energy (assuming 293.15 K), so that points at high energies are smaller.

In order to show in more detail the quality of the multipolar RESP fit, in Figure 4, we show on a grid-point level the correlation of the ESP from the reference BP86 calculation with the one approximated by DME2. For this figure, we generated a test grid different from the fitting grid, with 6482 points (shell radii between 1.0 and 2.5 times the vdW radii). In addition to the correlation of the ESPs, the plots also show the distances of the grid points from the nearest atom.

In the left column, all points are shown with an equal point size. It can be seen that most of the test grid points are close to the diagonal, indicating that the DME can reproduce the ESP of the electron density adequately. However, there are a significant number of grid points where the BP86 ESP is more positive than the ESP of the DME. These are mostly points close to the nuclei where the BP86 ESP is rather positive because of electron density penetration.

The right column of Figure 4 shows the same data except that the point size is scaled according to the Boltzmann weight (at 300 K) corresponding to the Lennard-Jones interaction energy of a hydrogen atom at the grid-point position. This suppresses all of the points that are close to the molecule (most points with dark color) and therefore less relevant for MD simulations. With very few exceptions, these relevant points show deviations of the ESP of less than ±0.1 eV (region between the black lines). This is true for all four electronic states, where no significant differences can be seen.

The deviations shown in Figure 4 can be summarized by computing the mean absolute deviations (MAD) and root-mean-square deviations (RMSD) across all points. The results are shown in Table 2. The values for the unweighted analysis are rather high, with MADs of about 80 meV and RMSDs of about 150–180 meV. However, the Boltzmann-weighted analysis shows significantly better results, with MADs of about 10 meV and RMSDs of about 15 meV. All electronic states can be reproduced at approximately the same accuracy. This is similarly true for the representation of the S_0_ →S_1_ and T_1_ →T_2_ transition densities, where we obtain Boltzmann-weighted MADs of 4 meV and RMSDs of 8 meV (see Figure S1 and Table S5). Hence, the DME representation of the electron densities seems to allow for a faithful description of the electrostatics around the molecule.

**Table 2:** Mean Absolute Deviations (MAD) and Root-Mean-Square Deviations (RMSD) of the Fitted DME with Respect to the BP86/def2-SVP Reference of the Distributions of Points in Figure 4 in eV

	unweighted		LJ weighted	
	MAD	RMSD	MAD	RMSD
S_0_	0.075	0.148	0.008	0.014
S_1_	0.082	0.180	0.008	0.013
T_1_	0.080	0.179	0.007	0.013
T_2_	0.080	0.151	0.009	0.016

### 4.3 Solvation Structure

Next, we compare the results for our electrostatic model of thioformaldehyde based on actual MD simulations with the reference electronic structure method and with LVC. Note that we only investigate the dynamics in S_0_ here, as a detailed discussion of the solvent-driven nonadiabatic dynamics of thioformaldehyde in water is beyond the scope of this work. For the S_0_ state, we computed four 1 ns trajectories (see above for details) and obtained 9502 snapshots from the DFT/MM trajectory and the same number from each of the three LVC/MM trajectories.

**Figure 5:**
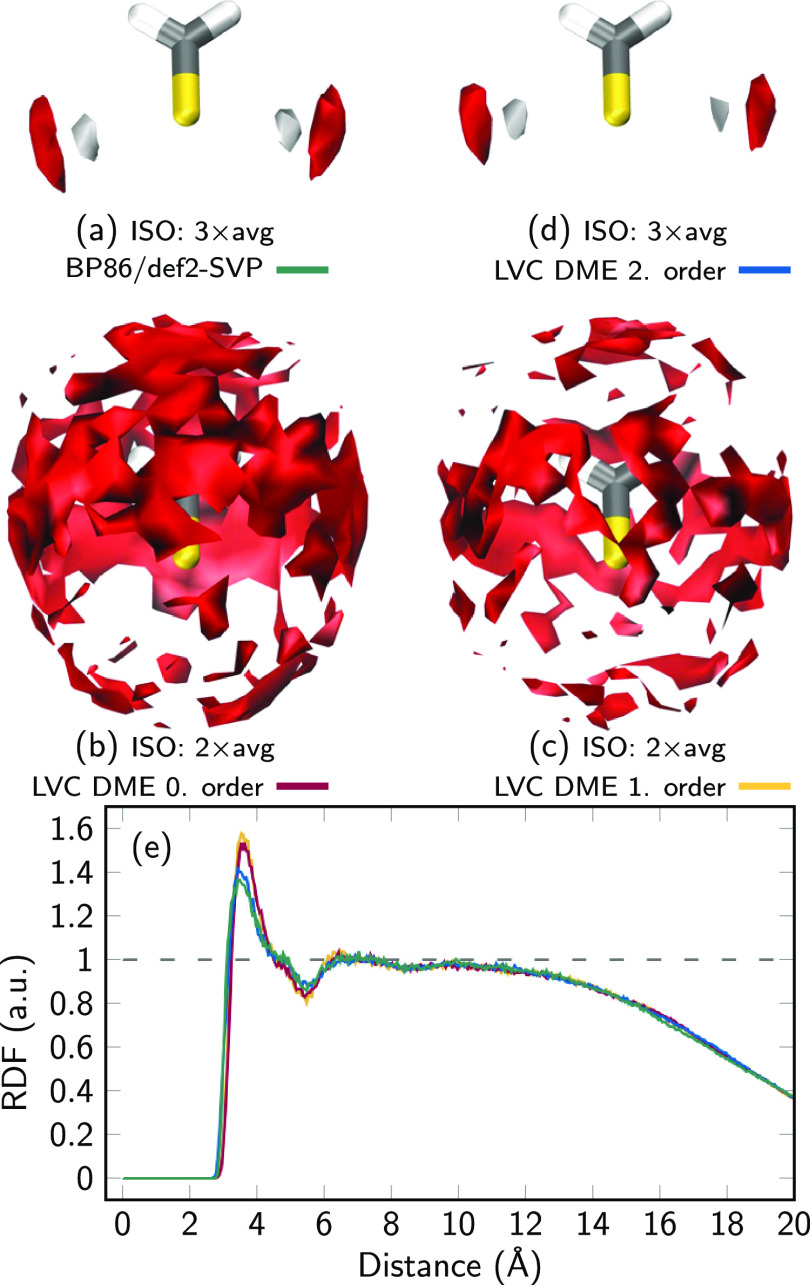
Solvent distribution of water around thioformaldehyde. (a–d) Isosurface plots (grid spacing 0.5 Å) of the most probable positions of water H atoms (white) and O atoms (red), with the isovalue specified as multiples of the average density in the box, for different levels of computation. Note the lower isovalues for panels (b and c). (e) Radial distribution functions (RDF) of water O atoms around the S atom with BP86/def2-SVP/TIP3P(green), LVC-DME0/TIP3P (red), LVC-DME1/TIP3P (orange), and LVC-DME2/TIP3P (blue).

The electrostatics around a molecule fundamentally influence the surrounding solvent molecules, leading to a particular solvation structure. The solvation structure of thioformaldehyde in the S_0_ state in water is presented in Figure 5. We first discuss the solvent distribution obtained from the 1 ns DFT/MM trajectory (panel a). As can be seen, thioformaldehyde forms two regions where it is very probable to find water H atoms (white) and likewise two larger regions of high-probability for O atoms (red). These regions are located in the *yz* molecular plane, orthogonal to the C–S bond (i.e., in *y* direction) around the S atom—clearly oriented toward the lone pair of the S atom. Therefore, it seems clear that the S atom lone pair forms distinct in-plane hydrogen bonds to water, which is well-known to occur for carbonyls.^76,77^ This shows very clearly that the solvation shell around the molecule is strongly anisotropic.

Figure 5b shows the 3D solvent distribution for the LVC-DME0/MM trajectory that only employs monopole charges. This calculation is thus equivalent to typical classical MM–MD calculations. As can be seen, this calculation produces a solvation structure very different from that of the DFT/MM calculation. Even at a lower isovalue, preferred H atom positions cannot be identified. The distribution of the O atoms is approximately isotropic around the molecule. Clearly, no directional hydrogen bonds are formed at this level of computation. Similar results are also obtained for the LVC-DME1/MM computation (panel c), which additionally employs dipole charges.

However, as shown in panel (d), the inclusion of quadrupole charges in the LVC-DME2/MM level of computation leads to an accurate reproduction of the anisotropic solvation pattern in thioformaldehyde, in good agreement with DFT/MM. This is a direct result of the minima of the ESP in the *xy* plane (Figure 2) obtained through quadrupole terms on the S atom. We note that there are still smaller differences between panels (a) and (d). For example, the hydrogen bonds in panel (a) are slightly stronger (with higher maximum occurrence) and form an angle of about 110° with the S–C bond, whereas in panel (d), the hydrogen bonds are slightly weaker and the angle is closer to 90°. This can be seen more easily in the difference plot in Figure S2.

Panel (e) of Figure 5 presents the obtained RDFs of the water O atoms around the S atom. The RDF obtained with DFT/MM shows a first maximum at about 3.5 Å, corresponding to the hydrogen bonds formed between S and water O and the remaining first solvation shell. At 5–6 Å, there is a slight minimum, indicating the region between the first and second solvation shells. At 7 Å, there is another very weak maximum, but generally, at this distance, there is little solvent structure. After 12 Å, the RDF starts to decrease due to the finite droplet that we are using.

The RDFs obtained with LVC-DME0/MM and LVC-DME1/MM produce a too strong first solvation shell peak but less hydrogen bonding at 2.8–3.4 Å, compared to DFT/MM. On the contrary, the LVC-DME2/MM calculation generally agrees very well with the reference in terms of the shape and locations of the maxima and minima. The first maximum at the LVC-DME2/MM level is only slightly stronger (by about 4%) than the reference RDF. We assume that this discrepancy is mostly due to the approximations inherent to the DME, i.e., using point multipoles instead of delocalized electron densities and using constant diabatic multipoles that neglect the geometry dependence and polarizability of the electron density. Apart from the maximum, the RDFs are fully consistent with each other at the obtained noise level.

At distances above 10 Å, where the RDFs begin to decrease, slight deviations can be observed between the different simulations. These deviations arise because the thioformaldehyde molecule does not stay sufficiently close to the origin and instead diffuses through the droplet in a different way in each of the trajectory. This indicates that the tether potential might have been chosen to be too weak to keep the molecule close to the origin. For the present investigations, we are confident that this is not a problem because the molecule never reached the surface of the droplet and was always surrounded by water. However, for future studies where the long-range behavior is important (e.g., X-ray scattering simulations), we would certainly recommend significantly stronger tether potentials. Furthermore, it might be good practice to crop cube or truncated octahedron droplets to spherical droplets for SHARC simulations like the one presented above because equilibration to a spherical droplet would take very long otherwise.

To also investigate dynamical properties, we computed hydrogen bond lifetimes from the DFT/MM and LVC-DME2/MM trajectory using a residence time approach.^78^ For this analysis, a hydrogen bond was defined by an O–S distance of <4.5 Å (see the main peak in the RDF in Figure 5e). The results are given in Figure S3, showing that both methods predict a biexponential hydrogen bond lifetime, although the ones from LVC-DME2/MM are larger by a factor of 2. We assume that the difference stems mostly from the missing polarizability of the employed single-state LVC-DME2 model. In models with multiple diabatic states, the interaction of the solvent with the respective transition densities is expected to recover the polarizability of the ground state to some degree, presumably increasing accuracy.

Furthermore, we analyzed the vibrational frequencies in the two simulations. We computed two new trajectories with identical settings, except saving coordinates every 0.5 fs between 5 and 10 ps and not constraining the C–H bond lengths. The normal-mode coordinates **Q** were subsequently Fourier-transformed. The results are shown in Figure S4 and Table S6. The vibrational spectra of the six normal modes agree reasonably well with each other. We assume that the differences (around 20 cm^–1^) are due to vibrational coupling to the water modes.

### 4.4 Potential and Solvation Energies

For completeness, we also investigated how well our LVC/MM approach can reproduce the total potential energies from the reference method. For this, we performed DFT/MM and LVC-DM2/MM single-point calculations for all 9502 snapshots described above. Additional DFT and LVC single-point calculations were performed for all 9502 snapshots after removal of the MM part. Therefore, our energy analysis is based on four sets of 9502 single-point calculations, each considering four states (S_0_, S_1_, T_1_, T_2_). For calculations including point charges, we deleted all MM energy contributions, keeping only the electronic energy of the molecule in the presence of the MM charges (term *E*_QM_(QM; MMpc) in eq 14). All energies are relative to the gas-phase equilibrium energy of BP86 or LVC, respectively.

**Figure 6:**
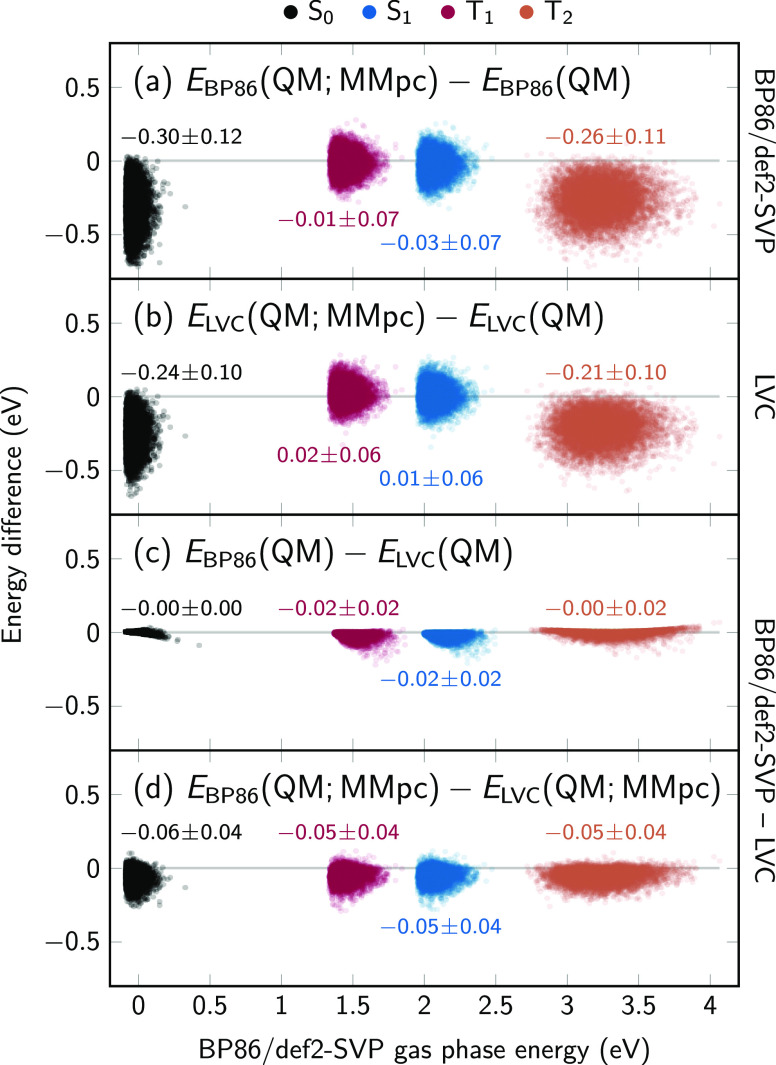
Scatter plots of energy differences computed at 9502 geometries. (a) Energy difference between BP86 calculation including point charges and BP86 without MM charges, i.e., electronic solvent stabilization energy. (b) Analogous energy difference using LVC-DME2/MM. (c) Energy difference between BP86 and LVC without MM charges, i.e., gas-phase agreement between the methods. (d) Energy difference between BP86/MM and LVC-DME2/MM, i.e., solution-phase agreement. The states are color-coded as S_0_ (black), S_1_ (blue), T_1_ (red), and T_2_ (orange). The mean values with standard deviations are included for each distribution (all in eV).

In Figure 6, we compare these four data sets by plotting various energy differences. In panel (a), we start with the difference between the BP86 energies including MM charges and those without MM charges. Thus, these energy differences represent the energetic stabilization of the respective states by the Coulomb interaction with the solvent charges. As can be seen, different electronic states are affected in different ways. The S_0_ state (black) is strongly stabilized by the interaction with the solvent because the considered snapshots were sampled from the trajectory in the S_0_; hence, the snapshots contain many solvent arrangements that are favorable for the S_0_ state. A similar picture is seen for the T_2_ state, which exhibits electrostatics very similar to those of the S_0_ state, as discussed above. On the contrary, the T_1_ and S_1_ states are not stabilized by the interaction with the solvent.

In panel (b), we show the same energy differences as in panel (a) but for the LVC/MM single-point calculations. It can be seen that the general trends of solvent stabilization (and the distribution of energies) are very well reproduced, with strong stabilization for S_0_ and *T*_2_, and little effect on T_1_ and S_1_. However, slight differences can be observed, especially in that the stabilization of S_0_ and T_2_ is systematically too small, by about 0.05 eV. This is consistent with the observations above that our DME charges predict slightly too weak hydrogen bonds and a slightly too shallow ESP.

In panels (c and d), we compare the two electronic structure methods directly, as it is not possible to compare the scatter plots in panels (a and b) point by point. Panel (c) shows the deviations between the gas-phase BP86 and LVC energies. Here, the agreement is excellent, with systematic and random errors on the order of 0.02 eV (0.5 kcal/mol), or even less for the S_0_ state. This shows that thioformaldehyde in the ground state can be accurately described by a harmonic oscillator potential, at least for geometries that are in the vicinity of the S_0_ minimum. This can be attributed to the rigidity of the molecule, i.e., the absence of low-frequency, strongly anharmonic, or curvilinear vibrational modes and the presence of only one ground-state minimum. This rigidity is a general prerequisite for the applicability of the LVC approach to any molecule.

In panel (d), we show the energetic deviations between the BP86/MM and LVC/MM calculations. The deviations are slightly larger than those in the gas phase, where the additional deviations are due to the imperfections of the DME in describing the true electrostatic interaction with the solvent. As mentioned above, the most relevant sources of errors here are the use of point multipoles and the neglect of the geometry dependence and polarizability of the electron density. However, we want to stress that the shown differences of around 0.05 ± 0.04 eV are equivalent to an error of 1–2 kcal/mol. If the systematic shift of about −0.05 eV across all states is ignored—as it will not affect dynamics, then the errors are about 1 kcal/mol. This is typically considered to be a desirable degree of accuracy in computing electronic energies. Hence, it appears that our LVC/MM approach using a DME can reproduce solvation energetics to a satisfactory degree, which is confirmed by the obtained solvent distributions shown above. Slight improvements might be possible by using different restraint parameters during the RESP fit, where the current restraints were chosen to be somewhat conservative to guard against overfitting.

### 4.5 Additional Remarks

As with many other model potential energy surface approaches,^21−24^ the accuracy of LVC/MM is bounded by the accuracy of the reference electronic structure method. Hence, a careful selection of the single- or multireference level of theory^79,80^ is of high importance. A particularly relevant aspect is whether the parametrization should be carried out with vacuum quantum chemistry or using implicit solvation treatment, where both approaches have their merits.

Parametrization in vacuum has been used extensively for various force fields,^81,82^ so LVC models used together with such force fields might be parametrized in vacuum, too, for consistency. Furthermore, LVC models with several diabatic states include some polarizability effects (from solvent-induced mixing of diabatic states)—in such models, parametrization should be done in vacuum to avoid double-counting of solvent-induced changes to charge distribution and geometry.^83^ A model parametrized in vacuum might also be transferable to different solvents if some polarizability is included. On the contrary, if the LVC model cannot be expected to provide sufficient polarizability (e.g., due to a too small diabatic basis), then it might make sense to parametrize using implicit solvation. This will provide multipole charges and geometries that are closer to the true condensed-phase ones.^83^

In the present case, parametrization was carried out in vacuum, but nearly identical parameters are obtained in implicit solvation. Repeating the parametrization using IEFPCM in water,^84^ the reference coordinates change by 0.001 Å, the frequencies by 14 cm^–1^, and the multipole parameters by at most 0.03 au (see Table S7). Thus, for thioformaldehyde, the solvation treatment during parametrization seems to make little difference, and already the vacuum parameters reproduce the solvation energies to 1 kcal/mol (Figure 6).

Significant improvements over the presented accuracy of the electrostatics (to further improve the agreement of LVC with its reference) would require additional implementations. The simplest addition would be to go beyond quadrupoles, including octupoles and potentially hexadecapoles. However, this would increase the number of parameters and thus the risk of overfitting significantly. Furthermore, it can be argued^85^ that multipoles higher than quadrupoles are not needed as long as all occupied or transition orbitals are predominantly composed of *s* and *p* functions. In order to improve the description of charge penetration, point multipoles could be replaced by Gaussian multipoles.^86,87^ The geometry dependence of the multipole charges (i.e., charge redistribution) could be further modeled by different charge flux models^34,88^ or more simply using linear dependence of multipole charges on normal modes. Further, polarizabilities could be treated (beyond solvent-induced mixing of diabatic states) using induced dipole models, Drude oscillators, etc.,^89−91^ possibly on both LVC and MM atoms. Finally, in addition to improving the description of the electrostatics, the LVC Hamiltonian itself can be improved by including higher-order terms in the Taylor expansion of **W** (eq 4). For example, quadratic and bilinear terms γ_*nn*_^(*i*)^ and γ_*nm*_^(*i*)^ would provide frequency shifts and Duschinsky rotations for the diabatic states.

We note that all of these additions would greatly increase the number of parameters, especially considering that our LVC/MM model is primarily intended for running nonadiabatic dynamics simulations using (possibly) a large number of electronic states. It is not clear whether one can robustly and efficiently obtain all of the necessary parameters for all electronic states and transition densities. Furthermore, these additions would increase the computational cost significantly, which might not be worthwhile unless the overall accuracy is significantly improved.

### 4.6 Computational Cost

For reference, we also briefly discuss the computational performance of our LVC/MM implementation. The four 1 ns trajectories discussed above were each computed on a single core of an Intel Xeon Gold-6234 16-core processor at a clock speed of 3.30 GHz. The DFT/MM trajectory using ORCA 5 was finished in approximately 240 h, giving a time of 1.72 s per time step. In contrast, each of the LVC/MM trajectories took 18 h, for a time of 0.13 s per time step. In all cases, approximately 0.06 s per time step is consumed by the MM calculation using OpenMM. The difference between the DFT and LVC calculations thus represents a speedup of more than an order of magnitude. We note that part of this speedup is due to several code optimizations that we carried out in the pysharc implementation^28^ of SHARC.

A speedup of an order of magnitude does not seem to be much considering that we compare a full-fledged electronic structure calculation including gradients with a simple harmonic oscillator and Coulomb scheme. However, we must note that thioformaldehyde in water is a very small test system (4 QM atoms and 3273 MM atoms), picked partially for its affordability to run a 1 ns ab initio QM/MM trajectory. For larger molecules or other electronic structure methods, the speedup of LVC/MM over QM/MM is expected to be vastly larger. We expect that the dominant cost in LVC/MM scales linearly in the number of LVC atoms and in the number of MM atoms as well as quadratically in the number of states. Only the numerical differentiation steps in the gradients (eqs 13 and 22) scale quadratically in the number of LVC atoms, but they are still very cheap. We confirmed the linear scaling with preliminary data from two other LVC/MM projects run in our group. There, another system with one state, 12 LVC atoms, and 8324 MM atoms took about 0.8 s per time step. A third system with one state, 61 LVC atoms, and 13500 MM atoms took roughly 4 s per time step. In contrast, the most efficient electronic structure methods scale at least cubically with the number of atoms (i.e., the number of basis functions). Many methods for excited states are even more expensive, whereas LVC/MM scales independently of the chosen reference method. Thus, it is easily conceivable that LVC/MM can be 3–5 orders of magnitude faster than QM/MM simulations, analogous to gas-phase LVC models.^14^

## 5 Conclusions

We have presented a complete framework for a hybrid linear vibronic coupling model electrostatically embedded into a molecular mechanics environment, termed LVC/MM. Within LVC/MM, multiple coupled potential energy surfaces of a solute molecule are described by using a reference harmonic oscillator and state-specific constant and linear coupling terms in a diabatic basis. We have shown how to extend LVC models to be rotationally and translationally invariant using the Kabsch algorithm to lift the restriction that the molecule must be aligned with its reference geometry. Subsequently, we introduced a Coulomb interaction matrix into LVC, whose matrix elements are computed from distributed multipole expansions for each diabatic electronic state/state pair. This allows for the computation of energies, gradients, and nonadiabatic coupling vectors for the entire LVC/MM system. Finally, we developed an extension of the restrained electrostatic potential fit technique to distributed multipoles.

The new LVC/MM method was implemented in the SHARC molecular dynamics package. The Kabsch algorithm and electrostatic embedding were incorporated into the SHARC-LVC interface, while the RESP fitting technique was implemented as a separate library that can be linked to any of the supported ab initio codes. We also implemented a general QM/MM interface and an OpenMM interface for SHARC. Finally, we added droplet and tether potentials to the SHARC dynamics driver and optimized the performance of the pysharc code and LVC interface.

The new method was applied to thioformaldehyde in water, which is a small test system that shows anisotropic solvation. By comparing to a 1 ns reference trajectory computed at the BP86/def2-SVP/MM level of theory, we show that LVC/MM can capture solvation effects that cannot be described with simple point charge models. Radial distribution functions were reproduced within a few % error and solvation energies within 1–2 kcal/mol. At the same time, LVC/MM provides a speedup of at least 1 order of magnitude over the reference DFT calculations and potentially much larger speedups for larger systems.

In summary, LVC/MM in SHARC provides a new approach to describe many picosecond nonadiabatic dynamics in solvated or embedded molecules at very low computational cost. The diabatic distributed multipole model is restricted to stiff and not too polarizable molecules in the same way that the underlying linear vibronic coupling model is restricted to stiff molecules. However, we believe that LVC/MM provides an unprecedented approach to describe the nonadiabatic dynamics of solvent molecules around an excited solute and offers unique opportunities for describing multichromophoric systems.

## A. Derivation of Multipolar RESP

RESP charges are fitted using a least-squares error function. The total error in the ESP *F*_error_^(*ij*)^ over all grid points **r**_*g*_, for a state pair *ij* and including only monopoles, is calculated as

31

The electrostatic potential *V*_esp_^(*ij*)^ at **r**_*g*_ is calculated with eq 17. The optimal charges *P*_*a*_^(*ij*)^ can be found by differentiating eq 31 with respect to the monopole charges and setting the result to zero

32

In the RESP model, eq 31 is modified with a hyperbolic penalty function

33

The charges *P*_*a*,ref_^(*ij*)^ are reference charges toward which the charges are restrained; in the entire manuscript, we generally assume all *P*_*a*,ref_^(*ij*)^ = 0 and omit them from all equations below.

The equation to find the optimal charges *P*_*a*_^(*ij*)^ can then be written as

34

After defining the restraining function *C* as

35

Equation 34 can be rearranged to

36

By further rearrangement, we can obtain

37

where we introduced the Kronecker delta to pull the constraint into the sum over *a*. We can now define

38

and

39

to write eq 37 in matrix notation

40

where **1** is a unit matrix and **C** is the vector collecting the restraint elements *C*(*P*_*a*′_^(*ij*)^).

The extension of the described RESP procedure to arbitrary multipole moments is rather straightforward by inserting eq 19 into eq 31. For clarity, we introduce the index *p* that enumerates the different multipole components (monopole, *x*, *y*, *z*, *xx*, *yy*, ···) and extend the total error by the multipole potentials

41

From this equation, one can derive the multipolar analogue of eq 37

42

By defining

43

and

44

and considering *a*′*p*′ and *ap* as composite indices, we end up at the same form of the matrix equation in eq 40.

## Supporting Information Available

Linear vibronic coupling parameters, raw parameter file contents, correlation plots for the RESP fit of transition densities, 3D difference solvent distribution plots, hydrogen bond lifetimes, vibrational frequencies from trajectories, and DME fits using implicit solvation (PDF)
