# Supplementary material for: LVC/MM: A Hybrid Linear Vibronic Coupling/Molecular Mechanics Model with Distributed Multipole-Based Electrostatic Embedding for Highly Efficient Surface Hopping Dynamics in Solution
Source: J Chem Theory Comput. 2023 Oct 3;19(20):7171–86. doi: 10.1021/acs.jctc.3c00805 (PMC10601485; doi:10.1021/acs.jctc.3c00805)
Supplement: Supplementary file 1 — ct3c00805_si_001.pdf [file ct3c00805_si_001.pdf]

Supporting Information: LVC/MM: A hybrid linear vibronic coupling/molecular mechanics model with distributed multipole-based electrostatic embedding for highly efficient surface hopping dynamics in solution

Severin Polonius,[†,‡] Oleksandra Zhuravel,[†] Brigitta Bachmair,[¶,‡] and Sebastian Mai[*,†,‡]

†*Institute of Theoretical Chemistry, Faculty of Chemistry, University of Vienna, Währinger Str. 17, 1090 Vienna, Austria.*

‡*University of Vienna, Vienna Doctoral School in Chemistry (DoSChem), Währinger Str. 42, 1090 Vienna, Austria.*

¶*Research Platform on Accelerating Photoreaction Discovery (ViRAPID), University of Vienna, Währinger Str. 17, 1090 Vienna, Austria.*

E-mail: sebastian.mai@univie.ac.at

Contents

S1 LVC/MM parameters for CH_2S S-2

S2 Raw LVC/MM parameters for CH_2S S-4

S3 Fits of the transition densities S-5

S4 Difference solvent structure plots S-6

S5 Hydrogen bond lifetime from trajectories S-7

S6 Vibrational spectra from trajectories S-8

S7 Parametrization in vacuum versus implicit solvation S-9

S1 LVC/MM parameters for CH_2S

All parameters of the LVC model are given in the following tables.

Tab. S1 presents the symmetries (in C_{2v}), frequencies, and assignment of the vibrational normal modes. Tab. S2 presents the ϵ parameters (vertical energy shifts). Tab. S3 presents the non-zero κ parameters (gradients in normal mode basis). Note that only totally symmetric modes can have non-zero κ parameters. Because all states have A_1 or A_2 symmetry, no two states of the same multiplicity have the same symmetry, and there are no A_2 modes, all λ parameters are zero.

Table S1: Vibrational normal modes of CH_2S calculated with BP86/def2-SVP.

Mode	Symmetry	Frequency (cm^{-1})	Description
7	B_1	967	asymmetric SCH bending mode
8	B_2	972	out-of-plane mode
9	A_1	1068	C-S stretching mode
10	A_1	1429	symmetric H-C-H bending mode
11	A_1	2965	symmetric C-H stretching mode
12	B_1	3053	asymmetric C-H stretching mode

Table S2: Values of ϵ parameters in eV.

State	Symmetry	ϵ
S_0	A_1	0.000
S_1	A_2	2.164
T_1	A_2	1.536
T_2	A_1	3.342

Table S3: Values of κ parameters in eV/Bohr.

State	Mode 9	Mode 10	Mode 11
S_1	0.141	−0.082	0.047
T_1	0.131	−0.092	0.062
T_2	0.397	−0.123	0.059

Tab. S4 on the next page provides the DME parameters (monopoles, dipoles, quadrupoles) for all four state densities (A_1 symmetry) and the two transition densities (A_2 symmetry).

Table S4: Values of DME parameters in e.

State	Atom	.	x	y	z	xx	yy	zz	xy	xz	yz
S_0	1C	−0.066	0.049	–	–	−0.071	0.053	0.018	–	–	–
	2S	−0.134	−0.064	–	–	0.390	−1.533	1.144	0.000	–	–
	3H	0.100	0.002	−0.011	–	−0.094	0.109	−0.015	0.082	–	–
	4H	0.100	0.002	0.011	–	−0.094	0.109	−0.015	−0.082	–	–
$S_0 \rightarrow S_1$	1C	–	–	–	–	–	–	–	–	–	0.205
	2S	–	–	–	–	–	–	–	–	–	−2.029
	3H	–	–	–	−0.118	–	–	–	–	0.046	0.199
	4H	–	–	–	0.118	–	–	–	–	−0.046	0.199
S_1	1C	−0.515	0.075	–	–	−0.002	0.003	−0.001	–	–	–
	2S	0.064	0.058	–	–	−0.024	1.182	−1.158	–	–	–
	3H	0.226	−0.064	0.073	–	0.146	−0.087	−0.059	−0.179	–	–
	4H	0.226	−0.064	−0.073	–	0.146	−0.087	−0.059	0.179	–	–
T_1	1C	−0.564	0.014	–	–	0.003	0.015	−0.019	–	–	–
	2S	0.101	0.083	–	–	−0.041	1.045	−1.004	–	–	–
	3H	0.232	−0.043	0.072	–	0.145	−0.068	−0.078	−0.212	–	–
	4H	0.232	−0.043	−0.072	–	0.145	−0.068	−0.078	0.212	–	–
$T_1 \rightarrow T_2$	1C	–	–	–	–	–	–	–	–	–	−0.514
	2S	–	–	–	–	–	–	–	–	−0.000	3.558
	3H	–	–	–	0.094	–	–	–	–	−0.227	−0.290
	4H	–	–	–	−0.094	–	–	–	–	0.227	−0.290
T_2	1C	0.178	−0.294	–	–	−0.077	0.070	0.007	–	–	–
	2S	−0.172	0.111	–	–	0.420	−1.631	1.211	0.000	–	–
	3H	−0.003	0.100	0.037	–	−0.071	0.132	−0.061	0.031	–	–
	4H	−0.003	0.100	−0.037	–	−0.071	0.132	−0.061	−0.031	–	–

S2 Raw LVC/MM parameters for CH_2S

Content of the input file `V0.txt`:

```
Geometry
 C   6.0  -1.95142000  -0.00000100   0.00000000  12.00000000
 S  16.0   1.11523600   0.00000300  -0.00000000  31.97207100
 H   1.0  -3.08108500  -1.76953400  -0.00000000   1.00782500
 H   1.0  -3.08109200   1.76952700  -0.00000000   1.00782500
Frequencies
  0.0000000000  0.0000000000  0.0000000000  0.0000000000  0.0000000000  0.0000000000  0.0044088473  0.0044298065  0.0048666679  0.0065116875  0.0135104928  0.0139108580
Mass-weighted normal modes
  0.0000000000  0.0000000000  0.0000000000  0.0000000000  0.0000000000  0.0000000000 -0.0000000000  0.0000000000  0.7306294482 -0.4021863573  0.2095454620 -0.0000000000
  0.0000000000  0.0000000000  0.0000000000  0.0000000000  0.0000000000  0.0000000000  0.3962132625 -0.0000000000  0.0000016191 -0.0000000000  0.0000000000  0.3304086934
  0.0000000000  0.0000000000  0.0000000000  0.0000000000  0.0000000000  0.0000000000  0.0000000000  0.4872934966 -0.0000000000  0.0000000000  0.0000000000  0.0000000000
  0.0000000000  0.0000000000  0.0000000000  0.0000000000  0.0000000000  0.0000000000  0.0000000000 -0.0000000000 -0.5480165125  0.0598310000 -0.0052199163  0.0000000000
  0.0000000000  0.0000000000  0.0000000000  0.0000000000  0.0000000000  0.0000000000 -0.1531275612  0.0000000000 -0.0000000000  0.0000000000 -0.0000000000 -0.0015816295
  0.0000000000  0.0000000000  0.0000000000  0.0000000000  0.0000000000  0.0000000000 -0.0000000000 -0.0802178308  0.0000000000 -0.0000000000  0.0000000000 -0.0000000000
  0.0000000000  0.0000000000  0.0000000000  0.0000000000  0.0000000000  0.0000000000  0.5885616191 -0.0000000000  0.2857954752  0.5254852136 -0.3470596724 -0.3536075788
  0.0000000000  0.0000000000  0.0000000000  0.0000000000  0.0000000000  0.0000000000 -0.2517488708  0.0000000000 -0.0353657818 -0.3757648771 -0.5979824186 -0.5660163166
  0.0000000000  0.0000000000  0.0000000000  0.0000000000  0.0000000000  0.0000000000 -0.0000000000 -0.6148618332  0.0000000000 -0.0000000000 -0.0000000000 -0.0000000000
  0.0000000000  0.0000000000  0.0000000000  0.0000000000  0.0000000000  0.0000000000 -0.5885625321  0.0000000000  0.2857935983  0.5254824504 -0.3470616318  0.3536104249
  0.0000000000  0.0000000000  0.0000000000  0.0000000000  0.0000000000  0.0000000000 -0.2517516097  0.0000000000  0.0353657818  0.3757667192  0.5979784999 -0.5660163166
  0.0000000000  0.0000000000  0.0000000000  0.0000000000  0.0000000000  0.0000000000 -0.0000000000 -0.6148618332  0.0000000000 -0.0000000000 -0.0000000000  0.0000000000
```

Content of the input file `LVC.template`:

```
V0.txt
2 0 2
epsilon
4
  1   1  0.0000000000
  1   2  0.0795144718
  3   1  0.0564322332
  3   2  0.1228161782
kappa
9
  1   2     9  5.17382e-03
  1   2    10 -3.01316e-03
  1   2    11  1.73842e-03
  3   1     9  4.81926e-03
  3   1    10 -3.39578e-03
  3   1    11  2.26771e-03
  3   2     9  1.45718e-02
  3   2    10 -4.52580e-03
  3   2    11  2.18598e-03
lambda
0
DMX R
 -6.1866055e-01  0.0000000e+00  0.0000000e+00  0.0000000e+00  0.0000000e+00  0.0000000e+00  0.0000000e+00  0.0000000e+00
  0.0000000e+00 -3.0959094e-01  0.0000000e+00  0.0000000e+00  0.0000000e+00  0.0000000e+00  0.0000000e+00  0.0000000e+00
  0.0000000e+00  0.0000000e+00 -2.1536359e-01 -0.0000000e+00  0.0000000e+00  0.0000000e+00  0.0000000e+00  0.0000000e+00
  0.0000000e+00  0.0000000e+00 -0.0000000e+00 -5.2621136e-01  0.0000000e+00  0.0000000e+00  0.0000000e+00  0.0000000e+00
  0.0000000e+00  0.0000000e+00  0.0000000e+00  0.0000000e+00 -2.1536359e-01 -0.0000000e+00  0.0000000e+00  0.0000000e+00
  0.0000000e+00  0.0000000e+00  0.0000000e+00  0.0000000e+00 -0.0000000e+00 -5.2621136e-01  0.0000000e+00  0.0000000e+00
  0.0000000e+00  0.0000000e+00  0.0000000e+00  0.0000000e+00  0.0000000e+00  0.0000000e+00 -2.1536359e-01 -0.0000000e+00
  0.0000000e+00  0.0000000e+00  0.0000000e+00  0.0000000e+00  0.0000000e+00  0.0000000e+00 -0.0000000e+00 -5.2621136e-01
Multipolar Density Fit (8x8x4x10)  settings [order grid firstlayer density layers] 2 lebedev 1.4 10.0 4
24
1  1  1  0    -0.06637323  0.04885895  0.00000015 -0.00000000 -0.07110716  0.05318751  0.01791964 -0.00000006  0.00000000 -0.00000000
1  1  1  1    -0.13391923 -0.06382663  0.00000012  0.00000000  0.38958044 -1.53337646  1.14379602  0.00000111 -0.00000000 -0.00000000
1  1  1  2     0.10014618  0.00195783 -0.01067182  0.00000000 -0.09358802  0.10871858 -0.01513056  0.08243313 -0.00000000  0.00000000
1  1  1  3     0.10014628  0.00195781  0.01067165 -0.00000000 -0.09358687  0.10871732 -0.01513045 -0.08243428  0.00000000  0.00000000
1  1  2  0     0.00000000  0.00000000 -0.00000000  0.00000010  0.00000000  0.00000000 -0.00000000  0.00000000  0.00000007  0.20530108
1  1  2  1    -0.00000000 -0.00000000 -0.00000000  0.00000015  0.00000000  0.00000000 -0.00000000  0.00000000  0.00000078 -2.02897635
1  1  2  2    -0.00000000  0.00000000  0.00000000 -0.11773070 -0.00000000 -0.00000000  0.00000000  0.00000000  0.04623295  0.19927442
1  1  2  3    -0.00000000 -0.00000000  0.00000000  0.11773042  0.00000000 -0.00000000 -0.00000000  0.00000000 -0.04623342  0.19927334
1  2  2  0    -0.51511634  0.07474811 -0.00000027 -0.00000000 -0.00181737  0.00327802 -0.00146065 -0.00000019 -0.00000000 -0.00000000
1  2  2  1     0.06379634  0.05812221 -0.00000014  0.00000000 -0.02416330  1.18180099 -1.15763769 -0.00000082  0.00000000 -0.00000000
1  2  2  2     0.22565995 -0.06366680  0.07342844  0.00000000  0.14623307 -0.08685768 -0.05937539 -0.17909362 -0.00000000  0.00000000
1  2  2  3     0.22566005 -0.06366632 -0.07342817 -0.00000000  0.14623174 -0.08685596 -0.05937578  0.17909527 -0.00000000 -0.00000000
3  1  1  0    -0.56432348  0.01365757 -0.00000037 -0.00000000  0.00341304  0.01515565 -0.01856869 -0.00000019 -0.00000000  0.00000000
3  1  1  1     0.10061170  0.08325909 -0.00000011  0.00000000 -0.04077352  1.04522095 -1.00444743 -0.00000081  0.00000000 -0.00000000
3  1  1  2     0.23185584 -0.04334201  0.07248726  0.00000000  0.14519531 -0.06756890 -0.07762641 -0.21209990  0.00000000 -0.00000000
3  1  1  3     0.23185595 -0.04334154 -0.07248692 -0.00000000  0.14519401 -0.06756721 -0.07762680  0.21210156 -0.00000000 -0.00000000
3  1  2  0     0.00000000  0.00000000 -0.00000000 -0.00000040 -0.00000000 -0.00000000  0.00000000  0.00000000 -0.00000023 -0.51381829
3  1  2  1    -0.00000000 -0.00000000  0.00000000 -0.00000003  0.00000000 -0.00000000  0.00000000 -0.00000000 -0.00000120  3.55814258
3  1  2  2     0.00000000  0.00000000 -0.00000000  0.09371261 -0.00000000  0.00000000 -0.00000000  0.00000000 -0.22738014 -0.29046039
3  1  2  3    -0.00000000 -0.00000000  0.00000000 -0.09371218  0.00000000 -0.00000000  0.00000000 -0.00000000  0.22738080 -0.29045836
3  2  2  0     0.17750475 -0.29430033 -0.00000033 -0.00000000 -0.07680620  0.07029024  0.00651596 -0.00000018  0.00000000  0.00000000
3  2  2  1    -0.17154526  0.11084784  0.00000039  0.00000000  0.41984779 -1.63094941  1.21110161  0.00000117 -0.00000000 -0.00000000
3  2  2  2    -0.00297973  0.10002803  0.03725977  0.00000000 -0.07100024  0.13222520 -0.06122496  0.03069275 -0.00000000  0.00000000
3  2  2  3    -0.00297976  0.10002835 -0.03725972  0.00000000 -0.07099948  0.13222429 -0.06122482 -0.03069355 -0.00000000 -0.00000000
```

S3 Fits of the transition densities

Fig. S1 together with Tab. S5 shows the quality of the fitted DME for the electronic transition densities between the singlet and triplet states, respectively. The figure is analogous to Fig. 4 From both Fig. S1 and Tab. S5 we infer that the DME represents the transition densities with the same accuracy as the state densities.

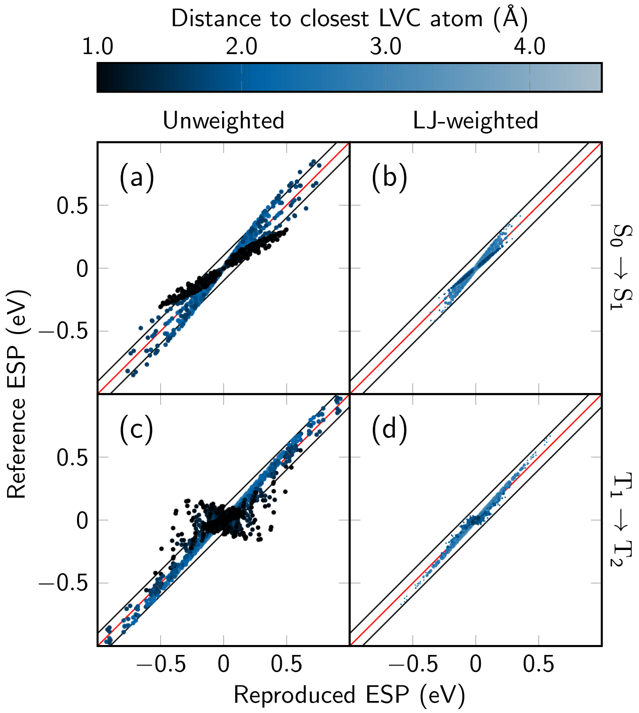

Figure S1: Correlation plots comparing the reference ESP energy (BP86/def2-SVP, vertical axes) with the ESP energy produced by the DME up to 2. order (horizontal axes). The plots use a test grid that is different from the fitting grid, with 6482 points in shells with radii of 1.0 to 2.5 times the vdW radii. The color of the points indicates the distance to the closest atom. The red and the two black lines indicate a deviation of zero and ±0.1 eV, respectively. On the left, all points are shown with equal point size, whereas on the right the point size is scaled by the Boltzmann weight of the Lennard-Jones interaction energy (assuming 293.15 K), so that points at high energies are smaller.

Table S5: Mean absolute deviations (MAD) and root-mean-square deviations (RMSD) of the fitted DME with respect to the BP86/def2-SVP reference of the distributions of points in Fig. S1 in eV.

	unweighted		LJ weighted	
	MAD	RMSD	MAD	RMSD
$S_0 \to S_1$	0.024	0.042	0.004	0.008
$T_1 \to T_2$	0.026	0.055	0.004	0.008

S4 Difference solvent structure plots

Fig. S2 shows the differences in three-dimensional solvent distributions obtained though simulations with different approximations to the electronic density. Panel (a) shows that the DME0 and DME1 calculations do not produce significant differences in solvent distribution (differences are below the isovalue of 1.5 time the average). In panels (b) to (e), the difference plots look similar to the DME2 and BP86 distributions (see Fig. 5b) and (c)). This is because the DME0 and DME1 distributions are quite isotropic, so subtracting them does not notably change the anisotropic distributions of DME2 and BP86.

From panel (f), we can see that DME2 and BP86 distributions differ slightly. Most notably, it can be seen that the positions of maximum solvent density are shifted—for DME2, the hydrogen bonds for an 90° angle with the C–S bond, whereas for BP86, the angle is closer to 110°.

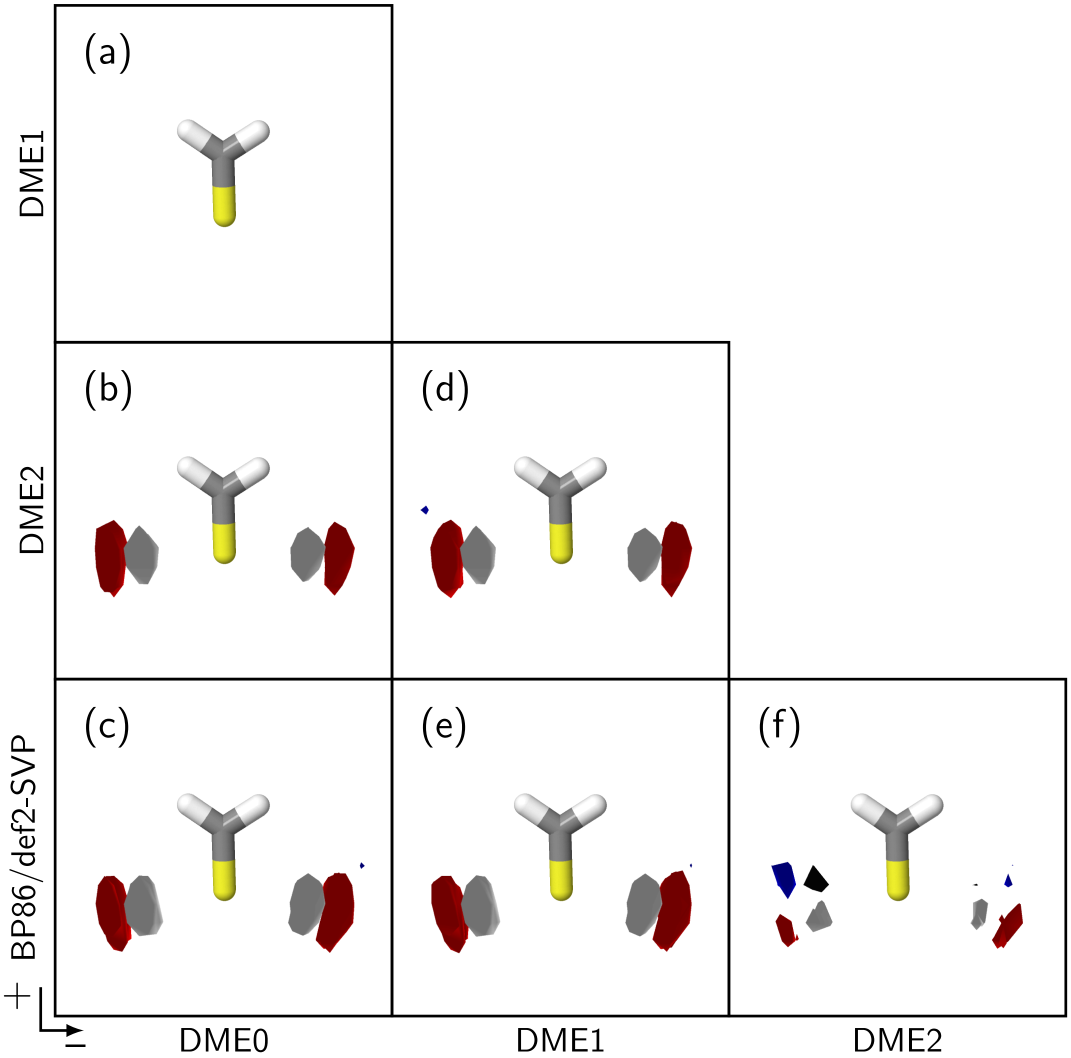

Figure S2: Differences in 3D solvent distributions resulting from the BP86/def2-SVP/TIP3P and LVC/TIP3P calculations with DMEs of different orders. The row and column labels refer 1 ns long simulations in the ground state with the respective method. Each panel ((a) to (f)) depicts the differences (row − column) of the three-dimensional histograms with occurrences over 1.5 times average colored in red (positive) and blue (negative) for oxygen, and white (positive) and black (negative) for hydrogen.

S5 Hydrogen bond lifetime from trajectories

Fig. S3 shows the distribution of residence times of O atoms within 4.5 Å of the S atom for both BP86/MM and LVC/MM simulations and their corresponding biexponential fits. The plots and fits show the typical behaviour of water that shows a short and a long residence time. We find that LVC/MM predicts residence times that are longer by a factor of two, compared to BP86/MM.

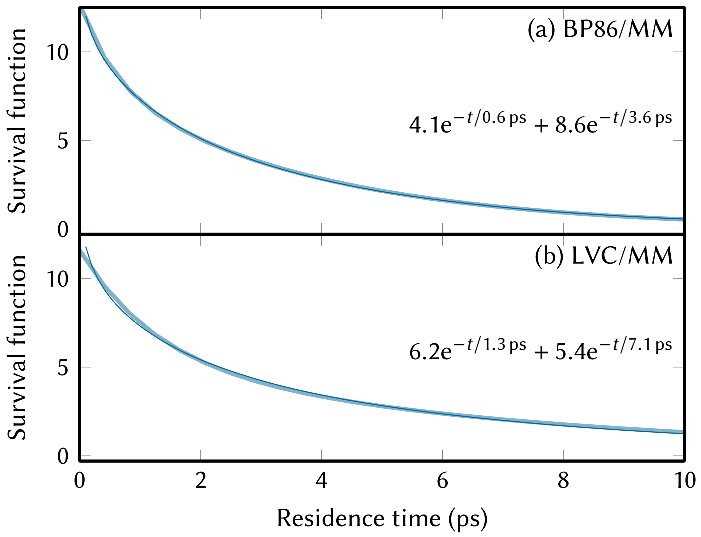

Figure S3: Water residence time plot, giving the distribution of residence times of O atoms within 4.5 Å of the S atom. Thin lines are the data, thick transparent lines are biexponential fits with the indicated functional form.

S6 Vibrational spectra from trajectories

Fig. S4 show vibrational spectra from two 10 ps trajectories with BP86/MM and LVC/MM. They were obtained from Fourier transformations of the time evolution of the six normal mode coordinates, sampled every 0.5 fs between 5 ps and 10 ps. Qualitatively good agreement between the two trajectories is found, with deviations of about 20 cm^{-1} (Tab. S1). We note that the frequencies of the LVC/MM trajectory differ slightly from the frequencies of the underlying harmonic oscillators.

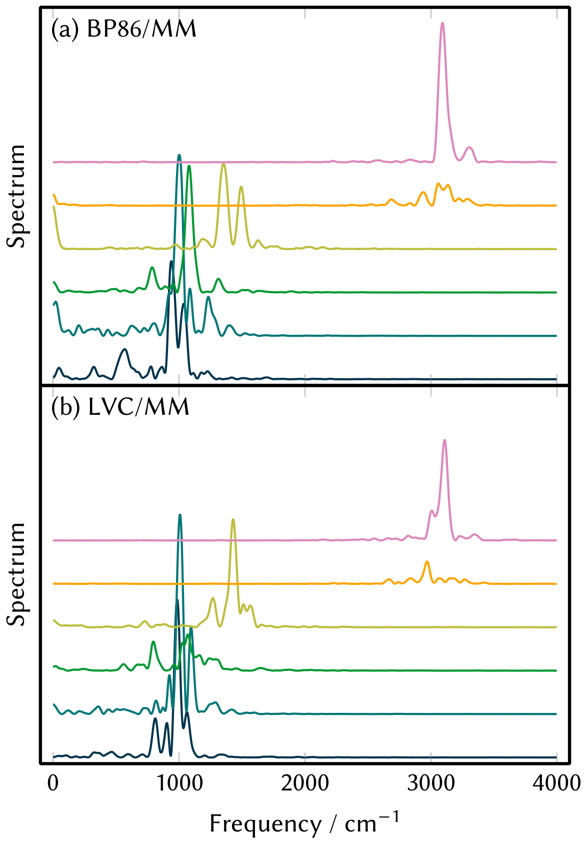

Figure S4: Vibrational spectra of thioformaldehyde obtained from Fourier transformation of the normal mode coordinates from two trajectories at for BP86/MM (a) and LVC/MM (b) level of theory. The plot shows mode 7 (bottom, blue) to mode 12 (top, pink), as defined in Tab. S1.

Table S6: Vibrational frequencies of thioformaldehyde from Hessian calculations and from BP86/MM and LVC/MM trajectories in cm^{-1}.

Normal mode	BP86 Hessian (vacuum)	BP86 Hessian (IEFPCM)	BP86/MM trajectory	LVC/MM trajectory
7	967	963	974	986
8	972	983	1003	1007
9	1068	1060	1078	1073
10	1429	1421	1410	1430
11	2965	2982	3030	3010
12	3053	3077	3090	3111

S7 Parametrization in vacuum versus implicit solvation

Tab. S7 shows the RMSDs of the different DME orders (monopoles, dipoles and quadrupoles) for every state and transition fitted to a calculation using IEFPCM compared to the used vacuum DMEs.

Table S7: RMSD (in their respective units e, ea_0, ea_0^2) for DME parameters obtained from vacuum and implicit solvation quantum chemistry, for the different orders (monopoles, dipoles, and quadrupoles).

Density	RMSD monopoles	RMSD dipoles	RSMD quadrupoles
S_0	0.033	0.004	0.006
S_1	0.011	0.003	0.007
T_1	0.011	0.001	0.008
T_2	0.021	0.014	0.013
$S_0 \rightarrow S_1$	0.000	0.001	0.002
$T_1 \rightarrow T_2$	0.000	0.000	0.016
